# Development of handling energy factors for use of dustiness data in exposure assessment modelling

**DOI:** 10.1093/annweh/wxae009

**Published:** 2024-02-24

**Authors:** Ana Sofia Fonseca, Carla Ribalta, Neeraj Shandilya, Wouter Fransman, Keld Alstrup Jensen

**Affiliations:** National Research Centre for the Working Environment (NRCWE), Lersø Parkallé 105, DK-2100 Copenhagen, Denmark; National Research Centre for the Working Environment (NRCWE), Lersø Parkallé 105, DK-2100 Copenhagen, Denmark; Federal Institute for Occupational Safety and Health (BAuA), Nöldnerstr. 40-42, 10317 Berlin, Germany; TNO, Risk Assessment of Products In Development, Utrechtseweg 48, 3704 HE Zeist, Netherlands; TNO, Risk Assessment of Products In Development, Utrechtseweg 48, 3704 HE Zeist, Netherlands; National Research Centre for the Working Environment (NRCWE), Lersø Parkallé 105, DK-2100 Copenhagen, Denmark

**Keywords:** continuous drop, dustiness, exposure assessment, exposure modelling, handling powders, nanomaterials, small rotating drum

## Abstract

Several exposure assessment models use dustiness as an input parameter for scaling or estimating exposure during powder handling. Use of different dustiness methods will result in considerable differences in the dustiness values as they are based on different emission generation principles. EN17199:2019 offers 4 different dustiness test methods considering different dust release scenarios (e.g. powder pouring, mixing and gentle agitation, and vibration). Conceptually, the dustiness value by a given method can be multiplied with a scenario-specific modifier, called a handling energy factor (*H*_*i*_), that allows conversion of a dustiness value to a release constant. Therefore, a *H*_*i*_, scaling the effective mechanical energy in the process to the energy supplied in the specific dustiness test, needs to be applied. To improve the accuracy in predictive exposure modelling, we derived experimental *H*_*i*_ to be used in exposure algorithms considering both the mass- and number-based dust release fraction determined by the EN17199-3 continuous drop (CD) and the EN17199-4 small rotating drum (SRD) test methods. Three materials were used to evaluate the relationship between dustiness and dust levels during pouring powder from different heights in a controlled environment.

The results showed increasing scatter and difference between the *H*_*i*_ derived for the 2 test methods with increasing pouring height. Nearly all the *H*_*i*_ values obtained for both SRD and CD were <1 indicating that the dustiness tests involved more energy input than the simulated pouring activity and consequently de-agglomeration and dust generation were higher. This effect was most pronounced in CD method showing that SRD mechanistically resembles more closely the powder pouring.

**What’s Important About This Paper?**

To move forward in human exposure assessment, there is an increasing need to use strong and validated predictive models. This study derives experimental handling energy factors as determined by the continuous drop and small rotating drum methods. These results will improve the exposure modelling of powder handling activities by helping to characterize dust generation.

## Introduction

Dustiness can be defined as the tendency of particles to become airborne from a powder in response to mechanical and/or aerodynamic stimulus (EN 15051; EN 17199 standards). The dustiness level and characteristics of dust release from a powder material can be related to different particle and powder characteristics (e.g. the primary particle/aggregate and agglomerate size distribution, surface-chemical modifications, relative density, and moisture content) as well as external parameters such as the agitation energy, and the relative humidity of the air in which the dust becomes airborne (Jensen et al. 2009; Schneider and Jensen 2009; Morgeneyer et al. 2013; Le Bihan et al. 2014; Levin et al. 2015; Jensen et al. 2016; Chakravarty et al. 2017a, 2017b; Shandilya et al. 2019). Measured dustiness data has been found to be a good potential predictor for estimating the emission potential of materials in powder form (Heitbrink et al. 1989; Breum et al. 2003; Ribalta et al. 2019a). Therefore, dustiness is used as an input parameter in several occupational exposure assessment models and tools for both nanomaterials and non-nanomaterials. Currently, at European level, only tools which were not developed nor tested, calibrated, and validated to address nano-specificity of materials are recommended in guidance documents by the European Chemicals Agency (ECHA) and accepted for use under the REACH regulation for risk assessment of general chemicals (e.g. Stoffenmanager, Advanced REACH Tool (ART) or ECETOC TRA; ECHA 2016). A number of new models, ranging from control banding to advanced aerosol dynamic models have been developed over the last years of which some are intentionally developed for exposure assessment of nanomaterials. Recently an analysis made by OECD showed that several of these tools showed a good performance for nanomaterials exposure assessment (e.g. Stoffenmanager Nano®, NanoSafer v1.1, and GUIDEnano tool; OECD 2021a, 2021b). Tools such as ART and Stoffenmanager incorporate a mechanistic source–receptor model and use dustiness ranking or absolute mass-based dustiness values (in mg kg^−1^) to scale exposure potentials (Marquart et al. 2008; Tielemans et al. 2008; Schinkel et al. 2011; Schneider et al. 2011). The control banding tool such as NanoSafer v1.1 use mass-based dustiness indexes to characterize the emission source strength (e.g. in mg s^−1^) so it can be used to estimate worker exposure (Kristensen et al. 2010; Jensen et al. 2014; OECD 2021a). In addition to the aforementioned tools, the use of tailored mass-balance models has been proposed and studied for worker exposure assessment by several authors in working environments (Koivisto et al. 2015, 2021; Arnold et al. 2017; Fonseca et al. 2019; Ribalta et al. 2021).

Different dustiness test methods exist and it is considered that good prediction of exposure using dustiness values should require a test method that adequately mimics the work process (Koivisto et al. 2015; Ribalta et al. 2019b and, 2019c). For example, the standard rotating drum (EN 17199-2:2019), and the small rotating drum (EN 17199-4:2019) methods were developed to simulate processes that involve repeated dropping, pouring, and agitation of powders, granulate materials, and the like, while the continuous drop (EN 17199-3:2019) method simulates powder pouring in a continuous feed. The vortex shaker (EN 17199-5:2019) is intended to simulate processes where the powder is subject to high-frequency vibration or shaking with high energy. Other currently non-standard methods, such as the venturi device, resembles a worst-case scenario comparable to the use of compressed air in cleaning activities (Evans et al. 2013; Palakurthi et al. 2022). Consequently, the dustiness data from different test methods will result in considerable differences in the absolute dustiness values as well as potentially different ranking orders (Jensen et al. 2016). Moreover, there are also differences in the actual energy by which the powders are agitated in the test methods and the agitation energies in the specific work processes. For example, the amplitude and frequency drop-heights, will in almost all cases differ between the ones used in the standard test methods and the release scenario in the workplace. Hence, if dustiness test data are used directly without modification in relation to the specific release scenario, the modelled exposure scaling and estimated emission rates will likely be very different from reality (Ribalta *et al.*, 2024). Therefore, a method-specific handling energy factor (*H*_*i*_), relating the mechanical energy in the release process to the energy applied in the specific dustiness test can be applied to enable more accurate exposure scaling or quantitative assessments (Schneider and Jensen 2008).

In NanoSafer, 4 precautionary *H*_*i*_ values were initially established conservatively considering the drop-height scaling developed for the ART. The requested dustiness data were intended to be produced using the rotating drum (RD; EN 15051-2:2013+A1:2016 or; EN 17199-2:2019) or the small rotating drum method (SRD; EN 17199-4:2019) which produce comparable results (shown in the EU FP6 NANODEVICE project, the pre-normative testing for the EN 17199:2019 standards and recently in an OECD inter-laboratory comparison; Ribalta et al., 2023). However, the original H_i_ values used in NanoSafer were reasonable worst-case estimates. Other studies have also used a handling energy factor to modify the dust emission potentials in quantitative mass-balance modelling (Koivisto et al. 2015). However, experimental values should be derived to improve the accuracy and generic use in exposure models using both mass-based and number-based dustiness data, which is facilitated by the EN 17199:2019 standards. Finally, it is desirable to enable exposure assessment using dustiness data generated by other methods such as the continuous drop (CD) test, as a different and highly relevant method in both EN 15051-3:2013 and EN 17199-3:2019 standards.

The purpose of this study was to derive experimental values for *H*_*i*_ to improve exposure modelling of powder handling activities considering both the number- and mass-based respirable, and inhalable dust release fraction as determined by the EN 17199-3:2019 CD and the EN EN 17199-4:2019 SRD methods. Preliminary results were used in an update of NanoSafer v.1.1beta on October 2, 2019, and following this publication values generated for EN 17199-3:2019 CD and minor adjustment of values derived for EN 17199-4:2019 SRD are implemented.

## Materials and methods

### Test design

**Figure 1.**
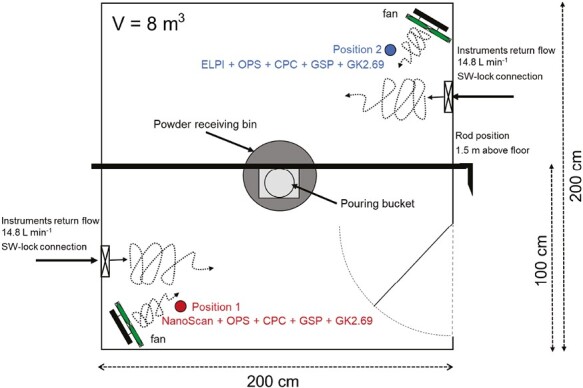
Principle design of the test chamber with indication of the pouring bucket and receiving bin, fans for air-mixing, and location of measurement instruments and samplers used.

A test design was established to determine the variation in *H*_*i*_ with increasing pouring height of powder in free air by comparison with dustiness test data. The pouring process was simulated in a test chamber allowing us to measure and characterize the aerosol release. The test chamber was 8 m^3^ in size (2 × 2 × 2 m inner dimension) and built to conduct the pouring tests at specific drop-heights in a turbulent mixed air-volume to make sure the aerosol instruments could detect small particle concentrations (Fig. 1). The test chamber was operated at low air-exchange rate; limited to volume-flow consumed by the external aerosol measurement devices (see below).

The dimension of the test chamber was made to match the near-field volume used in the NanoSafer v1.1 exposure assessment model. The chamber walls and ceiling were built in 15 mm polymethylmethacrylate plates mounted on an external wood-frame. The chamber was set inside a larger ventilated exposure chamber with stainless steel walls and floor, set at temperature and relative humidity controlled conditions (21 ± 3 ºC and 50 ± 5% RH) and 3–4 air exchanges h^−1^. All polymethylmethacrylate plate connections were sealed with tape and were connected and grounded with Cu-wires to reduce build-up of static electricity. Two small ventilation fans were placed in 2 opposite corners of the test chamber facing the centre to establish turbulent mixing of air (Fig. 1). Therefore, in this study it was assumed (and observed from real-time measurements) that the air with dust particles was fully mixed, and therefore the test chamber particle concentrations could be described with a mass balance of aerosol particles in a single compartment (Hewett and Ganser 2017).

Powder pouring was conducted from a steel bucket mounted on a plastic rod that could be rotated using a handle from the outside of the test chamber (Fig. 1). Photos of the test chamber, with visualization of the particle monitors and samplers used in each sampling position are shown in Supplementary Fig. S1.

### Sampling strategy and materials used

The measurement strategy adopted in this study aimed to simulate powder drop testing under controlled environmental conditions. It included real-time particle monitoring combined with a collection of samples for gravimetric analysis at 2 stationary near-field positions (Fig. 1). For background (BG) discrimination (i.e. particles from sources other than the target process), the non-experimental periods prior to the simulated drop tests were used to define the concentrations at the 2 measurement points. The inlets of the instruments and the samplers were approximately at the breathing zone height of 1.5 m and at a distance of 0.75 m from the center of the pouring bucket (diagonally positioned in corners in the chamber; Fig. 1 and Supplementary Fig. S1). Therefore, these 2 positions were representative of the average distance that a worker is assumed to be during a pouring process in an occupational environment. This sampling strategy allowed the estimation of concentration gradient, average room concentration, and finally total amount of emitted material during the simulated pouring activities. The pouring tests involved triplicate pouring of 3 different materials at 3 different open-air drop heights (25, 50, and 100 cm) and 2 different powder amounts (0.25 and 1 kg). The selection of these ranges of values was based on the rationale that they represent the range of the most common pouring activities in an occupational environment. In these experiments, the drop height corresponded to the distance between the bucket on the pouring rod and the bottom of the collection bin. The 25 cm drop height was the lowest practical possible drop height in the setup. The 100 cm drop height was considered as the maximum worst-case pouring height for this process and representing e.g., tank-filling operations and accidents. The 50 cm drop height was selected as an appropriate interim distance and a height normally not exceeded in powder pouring processes. Each triplicate test and consecutive dust sampling and real-time particle monitoring inside the test chamber had a duration between 15 and 33 min (Supplementary Table S1). Before preparation of a new test, the door from the chamber was opened to the surrounded ventilated exposure chamber and the room was not entered for refilling or cleaning until the total particle number concentration levels were satisfactory low and stable, between 500 and 1000 particles cm^−3^. The same concentration range was allowed at the start of new tests. The test chamber was vacuum cleaned between each triplicate test conducted for each drop height setting and amount. At the end of each set of powder experiments, the test chamber was also wiped with a damp soap-water cloth and dried with a damp pure water cloth.

The selected 3 materials were common pigments and fillers used for paint formulation: (1) modified alumino-silicate clay (OpTiMat^®^ 2550, Imerys, Barcelona, Spain); (2) calcined clay (PoleStar™ 200P, Imerys, Cornwall, United Kingdom); and (3) talc (Finntalc M15, Mondo Minerals B.V., Helsinki, Finland). Table 1 summarizes some of their physicochemical characteristics which were previously determined by Fonseca et al. (2021). All 3 materials had plate shapes, powder particle grain sizes *d*_*50*_ varying from 2 µm to 25 µm, bulk densities < 0.5 g cm^−3^, volume specific surface area (VSSA) <27 m^2^ cm^−3^ and moisture content <1.7% (Table 1). Even though none of these materials were identified as nanomaterials by the manufacturers, calcined clay PoleStar 200P is certainly classified as a nanomaterial as it has a plate shape and a VSSA >20 m^2^ cm^−3^ (European Union 2018). The other 2 materials can possibly also be classified as nanomaterials considering their plate shapes and uncertainty associated with the use of the VSSA method (Wohlleben et al. 2017) and the VSSA boundary of 6 m^2^ cm^−3^ defined in the revised EC definition of a nanomaterial (CIRS 2022). The study of Fonseca et al. (2021), showed that the pouring of both clays resulted in the release of nanometric particles into workplace air while emissions from the pouring of talc were dominated by coarser particles > 300 nm up to 7 µm.

**Table 1.** Description of physicochemical characteristics and dustiness of the materials under study.

Material name	*d*_*50*_ (µm)^¥^	Bulk density ± σ (g cm^−3^)^a^	VSSA ± σ (m^2^ cm^−3^)^b^	Moisture content ± σ (%)^c^	*DI*_*m-SRD*_ ± σ (mg kg^−1^)^d^	*DI*_*m-CD*_ ± σ (mg kg^−1^)^a^	*DI*_*m,inhalable-CD*_ ± σ (mg kg^−1^)^a^	*DI*_*N-SRD*_ ± σ (mg^−1^)^d^	*DI*_*N-CD*_ ± σ (mg^−1^)^a^	$\bar{S_{SRD}}$ × 10^6^ (min^-1^)^d^	$\bar{S_{CD}}$ × 10^6^ (min^-1^)^a^	MD1_SRD_/MD1_CD_ (µm)^e^	MD2_SRD_/MD2_CD_ (µm)^e^
Calcined clay (Al_2_Si_2_O_5_; PoleStar 200P; CAS No. 92704-41-1)	2	0.52 ± 0.01	27.0 ± 0.1	0.5 ± 0.1	13.3 ± 0.2	10.7 ± 1.5	117 ± 27	1189 ± 291	1381 ± 186	7.5 ± 2.0	5.7 ± 1.0	0.02/ 0.4	0.12/ 2.1
Talc (Mg_3_Si_4_O_10_(OH)_2_; Finntalc M15; CAS No. 14807-96-6)	5 (particles < 2 μm: 20%)	0.46 ± 0.01	15.1 ± 0.4	1.7 ± 0.4	69.1 ± 4.9	439.5 ± 44.7	7452 ± 3678	4610 ± 600	20293 ± 2017	28.8 ± 3.2	92.4 ± 11.1	0.02/ 0.26	2.0/ 2.5
Modified alumino-silicate clay (Al_2_Si_2_O_5_, OpTiMat 2550; CAS No. 93763-70-3)	25	0.16 ± 0.001	13.6 ± 10.4	0.2 ± 0.1	149.9 ± 10.8	394.5 ± 22.5	1790 ± 965	3312 ± 313	6333 ± 287	20.4 ± 1.4	18.3 ± 2.0	0.02/ 0.22	3.0/ 2.9

^¥^Information available in the material safety data sheets provided by the manufacturer. Median particle sizes were determined by using a gravitational liquid sedimentation method (ISO 13317-3 2001); ^a^Determined according to the procedure given in EN 17199-3:2019; ^b^Specific surface area determined by Brunauer–Emmett–Teller (BET) method with nitrogen absorption using an Autosorb-1-MP (Quantachrome, USA; (Macko and H. E. Emmett 1938)); ^c^Determined according to the procedure given in EN 17199-1:2019; ^d^Determined according to the procedure given in EN 17199-4:2019; ^e^Measured by SMPS (10 -500 nm) and APS, TSI (0.5 to 20 µm) in CD and ELPI (7-10 nm) in SRD.

σ: standard deviation; *d*_*50*_: median particle size; VSSA: volume specific surface area; *DI*_*m*_: respirable dustiness mass fraction; *DI*_*N*_: number-based dustiness index; SRD: small rotating drum method; CD: continuous drop method; $\bar{S}$: number-based average emission rate; MD1: number-size of the highest mode diameter measured; MD2: number-size of the second mode diameter.

These powder materials were pre-conditioned for 24 h and tested afterwards at controlled environmental conditions (21 ± 3 ºC and 50 ± 5 % RH).

### Particle monitoring and dust sampling techniques during the pouring tests

The measurements included real-time monitoring of particle concentrations and size distributions, and collection of particles on filter samplers simultaneously from 2 positions in the test chamber (Fig. 1 and Supplementary Fig. S1). The following real-time particle monitors were used:

- NanoScan (NS; TSI NanoScan model 3091, TSI Inc., Shoreview, MN, USA) to measure particle mobility size distributions from 10 to 420 nm in 60 s intervals (Tritscher et al. 2013; Fonseca et al. 2016).
- Optical particle sizer (OPS; TSI model 3330, TSI Inc., Shoreview, MN, United States) to measure the optical particle size distributions in 16 channels from 0.3 to 10 μm in 60 s intervals (Baron and Willeke 2001; McMurry 2002; TSI 2012).
- Electrical low-pressure impactor (ELPI; Dekati model ELPI+, Dekati Ltd., Kangasala, Finland) to measure the aerodynamic particle size distributions in 14 size channels between 7 nm and 10 µm with 1 s intervals (Keskinen et al. 1992).
- Portable condensation particle counter (CPC; TSI model 3007, TSI Inc., Shoreview, MN, United States) to measure the total particle number concentration from 10 nm to > 1 μm in 1 s time resolution (Matson et al. 2004; TSI 2007).

Diffusional losses for the NS and ELPI sampling lines were corrected according to Cheng (2001).

In the case of total particle number concentrations, we present the results obtained with the CPC, NS, and OPS because they are the instruments for which measurements were conducted at both positions in the test chamber. The ELPI data at position 2 needed to be discarded from data analysis due to its malfunctioning during the experiments. For this reason, only the results from the combined NS and OPS instruments at position 1 were used to present the particle number size distributions of the released emissions. Considering that the particles were well mixed in the chamber, small differences in particle size distributions at position 2 would be expected.

The mobility and optical particle number size distributions measured by the NS and OPS were combined to form a wide-size-range d*N*/dLog(*D*_*p*_) particle number size distribution according to Mølgaard et al. (2014). To make this combination it was assumed that a particle mobility and optical diameter were equivalent even though optical diameter may differ from mobility diameter depending on the particle shape, refractive index, and size (Pandis 2004). The NS last size channel (channel 15) was removed and the 14th channel was cut so that the upper boundary limit was the same as the OPS 1st size channel lower boundary limit (300 nm). Afterwards a new geometric mean diameter and channel width dLog(*D*_*p*_) values for the cut channel were calculated. The combined particle size distributions, named from here and onwards NSOPS, were based on the mobility size concentrations by NS from 10 to 300 nm and optical size concentrations by OPS from 300 nm to 10 µm.

The particle number size distribution was converted to mass size distribution by assuming that particles are spherical and the particle density does not vary with particle size. The density of each poured material was assumed constant. The mass size distribution covering the particle size range of 10 nm to 10 μm was converted to respirable mass size distribution by using the simplified respirable fraction penetration efficiency according to (Hinds 1999). It is assumed that the aerodynamic diameter and the optical diameter are the same.

The offline methods used in this study comprised collection of respirable dust (*d*_*50*_ cut size of 4 µm) and inhalable dust (*d*_*50*_ cut size of 100 µm) for gravimetric analysis by using Fluoropore (Millipore, Billerica, MA, United States) membrane filters 37-mm polytetrafluorethylene (PTFE) with a 0.8-μm pore size mounted in cyclones GK2.69 (BGI Inc., Waltham, MA, United States) or GSP-3.5 cyclone dust sampler (Sensidyne, LP, St. Petersburg, FL, United States), connected to portable sampling pumps (Apex2, Casella Inc., Bedford, United Kingdom) operating at 4.2 L min^−1^ and 3.5 L min^−1^, respectively (Stacey et al. 2014; Wippich et al. 2020).

Particle mass concentrations were gravimetrically determined by pre- and post-weighing the filters using an electronic microbalance (Mettler Toledo Model XP6) with ± 1 μg sensitivity located in a climate-controlled weighing room (relative humidity = 50% temperature = 22 °C). Three blind filters were stored to be used as laboratory blanks to correct handling and environmental factors.

### Dustiness characterization

The SRD method (EN 17199-4:2019) and the CD (EN 17199-3:2019) were used as standard techniques to determine the nanospecific dustiness data of the test materials. The SRD is designed to represent material handling at workplaces, including processes where bulk material is tipped, poured, mixed, scooped, dropped, or similar while the CD method simulates powder pouring in a continued feed. These methods are applicable to a wide range of materials including powders, granules, or pellets containing nano-objects in either unbound, bound uncoated and coated forms. As recommended in the standards, the pre-conditioning of powders (24 h) and the dustiness testing were conducted at temperature and relative-humidity controlled conditions (21 ± 3 ºC and 50 ± 5% RH).

The corresponding results presented in Supplementary Table S2 include a characterization of the inhalable dustiness mass fraction, respirable dustiness in terms of mass and particle number as well as the number-size distribution and the emission rates. Supplementary Annex I describes how the dustiness parameters were evaluated.

### Data treatment and calculations

#### Source term emission rate and handling energy factor

In NanoSafer v.1.1 control banding tool, the source term emission rate from process *i* by handling of powder *j* ($S_{i,j}$ in min^−1^ or mg min^-1^) is described and calculated from Equation. 1 (Schneider and Jensen 2008):


$$\begin{array}{l} S_{i,j}=DI_{j}\times H_{i}\times \frac{dM_{j}}{dt} \end{array}$$ (1)

where *DI*_*j*_ is the dustiness index of powder *j* expressed here in units of particle number (kg^−1^) or mass (mg kg^−1^), *H*_*i*_ (dimensionless) is the handling energy factor for the process *i* that is used to adjust the specific dustiness value to the release rate in the specific work scenario, and $\frac{dM_{j}}{dt}$ (kg min^−1^) is the powder *j* mass-flow in the transfer process. The *H*_*i*_, relates the mechanical energy used in the process *i* to the mechanical energy applied in dustiness index measurement. Thus, *H*_*i*_ can be expressed as:


$$\begin{array}{l} H_{i}=\frac{S_{i,j}\;\left(\;process\right)}{S_{i,j}\;\left(DI\;measurement\right)} \end{array}$$ (2)

Or


$$\begin{array}{l} H_{i}=\frac{\frac{Released\;M_{j}}{Handled\;M_{j}}}{DI_{j}} \end{array}$$ (3)

where released and handled *M*_*j*_ are the powder *j* mass released and handled in the process, and $S_{i,j}\;\;\;\left(\;process\right)$ and $S_{i,j}\;\;\;\left(DI\;\;\;measurement\right)$ are the source term emission rate from process *i* by handling of powder *j* obtained in the actual handling process and in the dustiness experiment, respectively.

The *H*_*i*_ of the process equals 1 if the applied mechanical energy equals the energy that was used to measure the dustiness index or has the same effective dust generation and dispersion effect. If de-agglomeration was complete in the dustiness index measurement, then 0< *H*_*i*_ < 1 when measuring dustiness in particle numbers. If de-agglomeration and dust generation were not complete in the dustiness test, *H*_*i*_ could be > 1.

#### Particle dynamics

Assuming that concentrations are fully mixed in the dustiness system and during the simulated handling process, the source term emission rates *S* (s^−1^ or mg s^−1^), can be calculated by using a governing mass balance equation of aerosol particles in a single compartment (Equations 4 and 5) previously described by several authors and in the standard EN 17199-4 (EN 17199-4:2019; Schneider and Jensen, 2008; Jensen et al., 2009; Hewett and Ganser, 2017):


$$\begin{array}{l} V\cdot \frac{dC\;\left(t\right)}{dt}=S\;\left(t\right)-QC\;\left(t\right) \end{array}$$ (4)

$$\begin{array}{l} S\;\left(t\right)=V\frac{dC\;\left(t\right)}{dt}+QC\;\left(t\right) \end{array}$$ (5)

where d*C*/d*t* is the change in background-corrected particle concentration [cm^−3^·s^−1^ or mg m^−3^·s^−1^], *V* is the volume of the dustiness system or room chamber (in cm^3^), *t* is the time, and *Q* is the volume flow rate in the room or through the dustiness system (in cm^3^ s^−1^). *Q* is related to the exponential particle decay rate, *γ* (in s^−1^) by Equation 6 representing the total particle loss rate, including, e.g. particle removal from deposition losses in tubing and on surfaces.


$$\begin{array}{l} Q=V\cdot \gamma \end{array}$$ (6)

The particle losses are calculated on a fit from the recorded particle concentration, which may be dependent on the mixing state of the dustiness system or chamber and the characteristics of the powder used, as powder with larger particles will have a higher gravimetric deposition rate. When the particle emissions are negligible (i.e. *S*(*t*) ≈ 0 s^−1^) the particle number concentration decay curve may be described as follows:


$$\begin{array}{l} C\;\left(t\right)=C_{t=0}e^{-\gamma \cdot t} \end{array}$$ (7)

According to the convolution theorem, the particle number concentration during the simulated activity and dustiness tests is a convolution of the particle sources and particle losses where the particle emission term can be solved with a numerical deconvolution as follows (Schripp *et al.* 2008):


$$\begin{array}{l} S\;\left(t\right)=V\frac{C\;\left(t\right)-C\;\left(t-△t\right)\cdot e^{-\gamma \cdot △t}}{△\;t} \end{array}$$ (8)

In this work, the particle number emission rates (s^−1^) were calculated for the BG period when *S*(*t*) = 0 s^−1^ and subsequently subtracted from the total particle generation rate during the pouring drop or the dustiness test.

#### Statistical analysis

The Minitab 17 program (v. 17.1.0; Minitab Ltd, UK) was used to perform the statistical analyses. The analyses comprised outlier analysis (using Grubb’s test), statistical distribution analysis, descriptive statistics, regression analysis, and final derivation of an overarching function for *H*_*i*_ versus drop height. All calculations were made using the default functions in the software. Microsoft Excel was used for additional plotting and comparative calculations of means, medians, and quartiles. Only minor differences <3.7% were observed between Minitab and MS Excel values.

## Results

### Dustiness characterization

A summary of the dustiness data generated for all the materials by using the SRD and CD can be found in Table 1. According to the SRD, the respirable dustiness mass-fractions of all the 3 materials were relatively low with clay OpTiMat having the highest (150 ± 11 mg kg^−1^) and calcined clay PoleStar having the lowest dustiness value (13 ± 0.2 mg kg^−1^). According to the EN 15051-2 ranking system established for the RD, clay OpTiMat and talc are in the category of powders with moderate (between 60 and 210 mg kg^−1^), and clay PoleStar is close to very low dustiness level (< 10 mg kg^−1^). Contrarily, the talc material had the highest number-based dustiness index by using both the SRD method and CD methods. Also, the respirable and inhalable dustiness mass-fractions were highest for talc (440 ± 45 mg kg^−1^), followed by clay OpTiMat and calcined clay PoleStar in the CD method. Considering the EN 15051-3 ranking system established for the CD method, the respirable dustiness mass-fractions obtained for talc and clay OpTiMat fall into the category of high dustiness (>300 mg kg^−1^) while calcined clay PoleStar into the very low dustiness level (<20 mg kg^−1^).

The evidence for clay OpTiMat and talc being the dustiest materials in the dustiness tests can be supported by Supplementary Fig. S2, which clearly shows different particle generation rate time profiles and kinetic data obtained with the SRD and CD methods. While a brief initial burst was observed for both talc and calcined clay PoleStar materials, a slowly increasing particle generation rate reaching a plateau towards the end of the dustiness test was verified for clay OpTiMat. However, the talc had a significantly higher emission rate in the CD than the SRD (3.2 times higher) (Table 1). This difference might be due to several mechanisms like de-agglomeration. Here, the associated energy input used in the CD method may have contributed to the highest level of de-agglomeration (i.e. break-up the agglomerates) and dispersion of the powder talc particles due to agglomerate-agglomerate collision or impacts against the wall thus generating aerosols with a highest particle number and mass concentrations (Ding et al. 2015).

The corresponding particle number size distributions and the 2 highest mode diameters confirmed that the dustiness tests by SRD and CD methods of both talc and clay OpTiMat released coarser particles and consequently, higher respirable dustiness mass-fraction and number-based dustiness index (Supplementary Fig. S3 and Supplementary Table S2).

With caution, we do not have the primary particle size, it is observed that the mean particle size and VSSA of the powders have an influence on the dustiness. The low dustiness for the finest material (clay Polestar; *d*_*50*_ = 2 µm and *VSSA* = 27 m^2^ cm^−3^) may be due to stronger Van-der-Waals forces for the small particles. On the other hand, the low dustiness of the coarsest material (clay OpTiMat; *d*_50_ = 25 µm and *VSSA* = 13.6 m^2^ cm^−3^) could possibly be explained by higher mass due to size (Chakravarty 2018).

### Particle concentrations and emission rates

**Figure 2.**
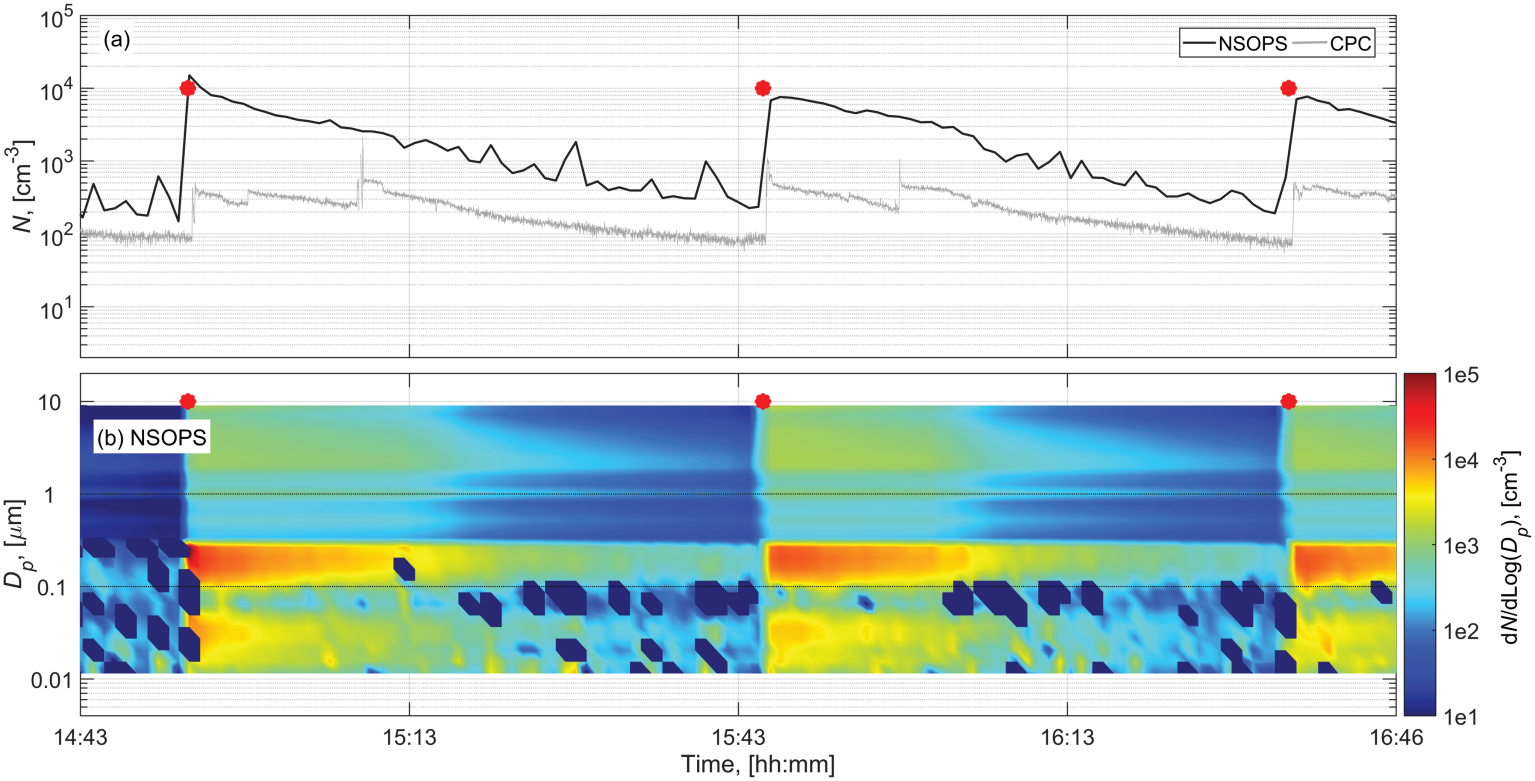
Time series data obtained during pouring of 1 kg of clay OpTiMat at the height of 1 m: (a) particle number concentrations measured by CPC and NSOPS from position 1, (b) particle number size distributions measured from position 1 by using the NSOPS. The symbols shown as asterisk represent the pouring times (3 replicates).

The results of the particle number concentration measurements from the pouring tests were analyzed considering 2 periods: background (5 min before the drop tests), and pouring activity. The process-specific average total number and mass concentrations obtained in positions 1 and 2 are shown in Supplementary Table S1. Figure 2 shows an example of a time series of the recorded particle number concentration and size distributions while pouring the dustiest material of clay OptTiMat (according to the SRD) at the highest drop height of 1 m and largest amount of material, 1 kg. Concentrations measured at position 2 were omitted from the figures because similar particle number concentration patterns were obtained. Mean particle number concentrations measured by CPC at position 1 varied from 61 to 1200 cm^−3^ which were on average 1.2 times higher than the concentrations measured at position 2 by using a similar instrument (see Supplementary Table S1). Differences below 30% were also obtained in more than 75% of filters collected in both positions for either respirable or inhalable mass (Supplementary Table S1). This indicates that particles were well mixed at all times in the chamber. According to the online instruments, the highest particle number concentration was measured by NSOPS while pouring 1 kg of talc from 1 m height (2.1 × 10^4^ cm^−3^) which corresponded to the material with the highest respirable dustiness fractions in terms of particle number by using the SRD and CD dustiness methods. On the other hand, similarly to the results from SRD dustiness test, the pouring of 1 kg of clay OpTiMat from 1 m height was the activity which released more respirable and inhalable particles (average *PM*_*4*_ and *PM*_*inhalable*_ from position 1 and 2 = 10.4 and 63.5 mg m^−3^, respectively; Supplementary Table S1).

**Figure 3.**
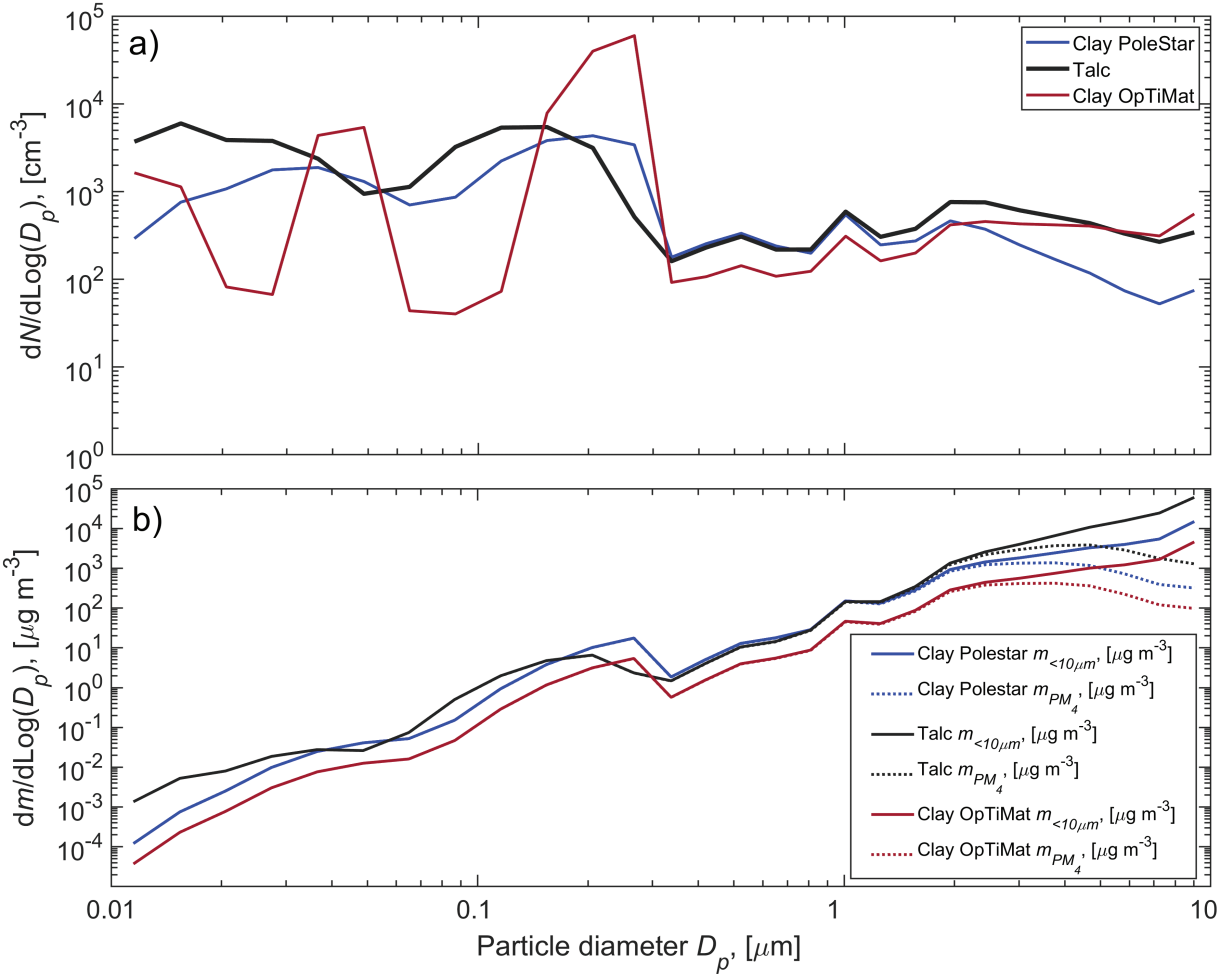
Averages of (a) particle number size distributions and (b) mass size distributions for concentrations measured at position 1 by the NSOPS while pouring 1 kg of material at the highest drop height of 1 m.

The particle number size distributions measured during the pouring tests of talc material confirmed a more pronounced release of fine particles while the clay OpTiMat emitted coarser particles (Fig. 3). This fact explains the highest levels of particle number and mass concentrations measured when talc and clay OpTiMat materials are poured, respectively.

Supplementary Fig. S4 clearly shows that the higher the mass flow and pouring height, the higher the particle emissions. For example, during pouring 1 kg of clay OpTiMat from 1 m height, the particle number and respirable mass released were 6.4 and 2.4 times higher than during pouring 0.25 kg, respectively. At a constant mass of dropped material, the number and mass of particles released from 1 m were up to 20 times higher than when dropping from 25 cm.

Independently on the material used in the pouring tests, the particle number concentration levels decreased exponentially immediately after the activity (Supplementary Figs. S5, S6, and S7). These periods were used to estimate the decay rates for particles measured by the online instrumentation for each drop test material by using Equation 7. An average decay rate, *γ* of 2.5, 3.4, and 4.5 h^−1^ was used in Equation 8 to determine the particle number emission rates for clay PoleStar, talc, and clay OpTiMat, respectively.

As expected, similarly with the real-time particle number concentration, the highest background corrected particle number emission rates were obtained while pouring the talc, which released the smallest particle sizes (Supplementary Table S2). The particle emission rate was 8.0 × 10^9^ min^−1^ when 1 kg of material was poured from 1 m height.

### Test-specific handling energy factors

The handling energy factors (*H*_*i*_) were calculated for each pouring test considering the particle number emission rates and mean respirable and inhalable dustiness mass fractions (Supplementary Table S3). The H_i_ calculated for number, respirable mass, and inhalable mass varied from 0 to 3.8, 0.003 to 0.7, and 0.002 to 0.15, respectively. The highest *H*_i_ defined for particle number emission rates were obtained for clay polestar in test 4 and clay OpTiMat in test 17 (≥1) meaning that the agitation energy experienced during these pouring tests was similar or higher than the energy inducted during the dustiness tests. Conversely, for all the other tests, a complete or higher de-agglomeration might have occurred in the corresponding dustiness experiments as the *H*_*i*_ for particle number was <0.9. Handling energy factors <0.7 were determined for respirable and inhalable mass for both CD and SRD dustiness methods. While talc was found to be the material with highest respirable mass-based *H*_*i*_ for SRD, the clays Polestar and OpTiMat were the materials with the highest *H*_*i*_ in CD for respirable and inhalable fractions.

Based on the individual *H*_*i*_ values calculated for each experiment above, a statistical analysis was made on the relationship between the test parameters (pouring height, amount poured, VSSA, and dustiness index) and the derived *H*_*i*_ values to establish an overarching relationship between test parameters and *H*_*i*_ to be used for modelling. All individual *H*_*i*_ values calculated were included in an outlier analysis followed by principal component analysis, a statistical distribution analysis, regression analysis between principal components, and selection of *H*_*i*_ values to establish a function to derive the final *H*_*i*_ values for use in exposure assessment modelling.

As a first step, an outlier test of *H*_*i*_ for both SRD and CD dustiness methods considering a normal distribution was made for all *H*_*i*_ values derived for each pouring height to assess whether all data should be included in the data set for further statistical analysis. The results in Supplementary Table S4 showed only one outlier in the *H*_*i*_ values, which was the *H*_*i*_ for the 25 cm pouring height compared with the particle number-based DI considering normal distribution and a 95% confidence interval (*P*-value < *α* meaning that there is evidence to conclude that an outlier exists).

A standard descriptive statistical analysis was made on the calculated *H*_*i*_ values for both the SRD and CD methods. The results of this analysis presented in Supplementary Table S5 revealed that the *H*_*i*_ values at each pouring height can be considered to follow a weakly to clearly skewed normal distribution. Box plots of the *H*_*i*_ values are plotted in for each of the pouring heights tested (Supplementary Fig. S8). A highly upper 3^rd^ quartile (Q3) skewed distribution is observed at 100 cm pouring height, especially for CD method (respirable and inhalable mass; Supplementary Fig. S8b and S8e).

The multivariate principal component analysis (PCA) shows a clear correlation between *H*_*i*_ and drop height while a low-power opposite correlation was found to dustiness indexes and VSSA. Additionally, a poor correlation with the amount of material poured was found (Supplementary Fig. S9). Based on the PCA analysis, regression analysis was first made between *H*_*i*_ and the pouring height. A high correlation was found already for linear regression analysis (*P*-value < 0.001). However, analysis of the residuals shows a considerable scatter for *H*_*i*_ values determined by dustiness mass fractions (*R*^2^ < 59.7%) and number-based dustiness indexes (*R*^2^ < 3.1%) which becomes important with increasing pouring height (data not shown). The residual plots for the linear regression plot suggest an abnormal distribution for some of the individual *H*_*i*_ values calculated for the SRD and CD. This atypical distribution may be caused by the influence of secondary parameters on *H*_*i*_. Performing a multiparametric linear regression analysis (Supplementary Figs. S10–S14) showed that the addition of the dustiness indexes, VSSA, and the amount poured as a parameter to the equation, improved the predictability due especially to the negative correlation with the dustiness indexes and VSSA (*R*^2^ = 80% and 20% for *H*_*i*_ values determined by dustiness mass fractions and number based dustiness indexes, respectively).

## Discussion

**Figure 4.**
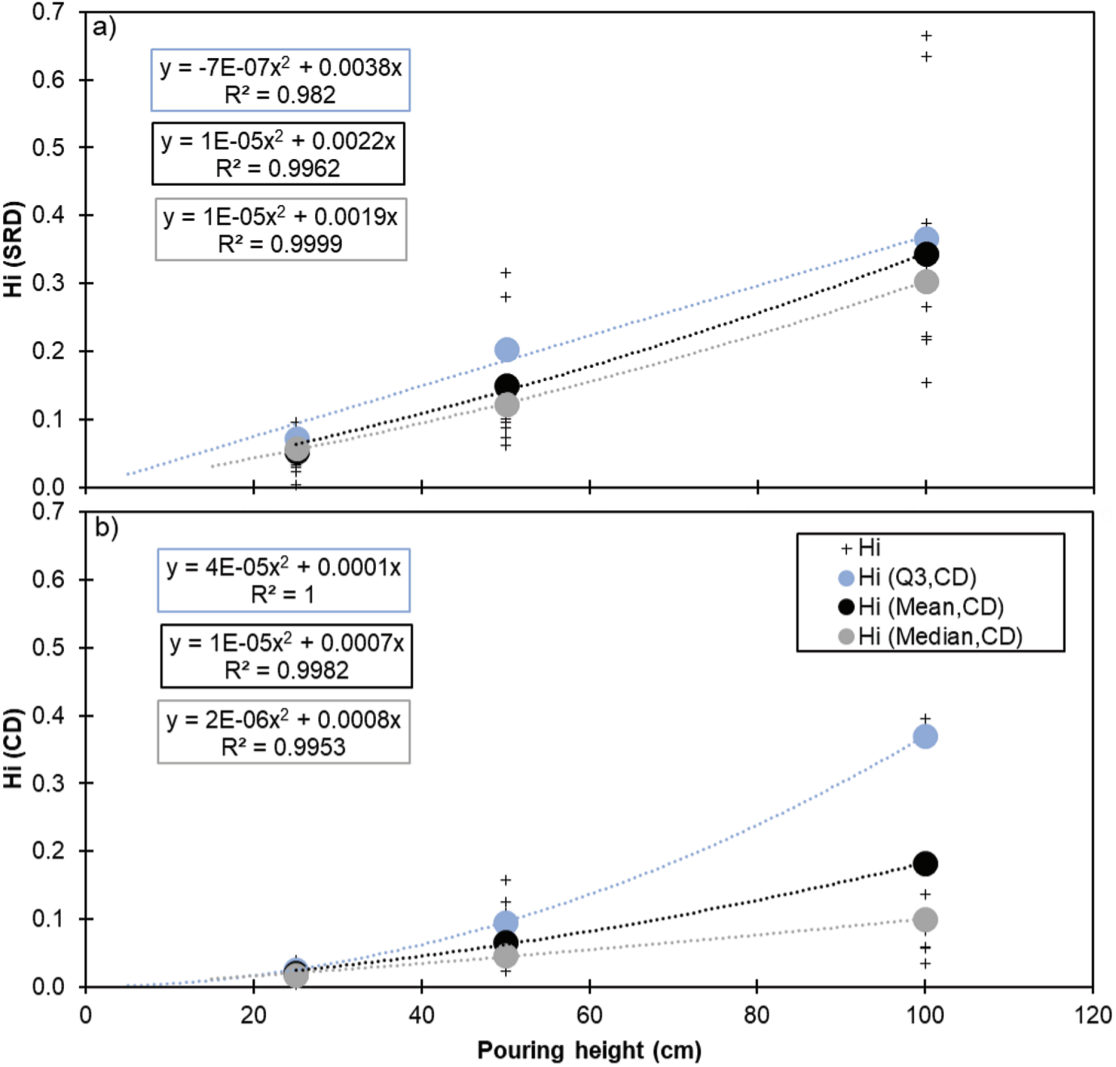
Individual calculated *H*_*i*_ values (determined by respirable dustiness mass fraction), regression curves, and functions for the mean, median, and upper 3^rd^ quartile (*Q*3) values for SRD (a) and CD dustiness method (b).

Analyzing the correlations between all parameters and the *H*_*i*_ calculated for each drop test experiment, the drop height is the variable with highest influence. However, due to the scatter observed around the mean value with increasing drop height, the uncertainty and risk of under-prediction in the exposure assessment are considered significant. Statistically, there is 7% and 103% difference between the mean and the Q3 of the *H*_*i*_ determined by the respirable dustiness mass fractions at 100 cm pouring height for SRD and CD, respectively (see Fig. 4). As for the *H*_*i*_ determined by the particle number concentrations, differences of 62% and 54% were registered for the SRD and CD, respectively (Supplementary Fig. S15). Lastly, a difference of 38% was registered for *H*_*i*_ determined by the inhalable dustiness mass fraction obtained for CD dustiness method (Supplementary Fig. S16). Therefore, for precautionary reasons, it was decided to establish a general regression function based on the Q3 of the individual calculated *H*_*i*_ values. The regression was forced through (0,0) and the resulting equations were used to calculate the specific *H*_*i*_ values at given heights to be used in the NanoSafer exposure assessment model considering an approximate doubling in *H*_*i*_ between each step when considering the mass and number-based dustiness indexes (Table 2).

**Table 2.** Calculated *H*_*i*_ values, standard deviations (*σ*) and their increment ratios as function of pouring height considering the upper 3^rd^ quartile (Q3).

Dropping height (cm)	Determined by *DI*_*m (respirable)*_				Determined by *DI*_*N*_				Determined by *DI*_*m (inhalable)*_	
	*H*_*i,SRD*_ ± σ	*H*_*i,SRD*_/*H*_*i-1,SRD*_	*H*_*i,CD*_ ± σ	*H*_*i,CD*_/*H*_*i-1,CD*_	*H*_*i,SRD*_ ± σ	*H*_*i,SRD*_/*H*_*i-1,SRD*_	*H*_*i,CD*_ ± σ	*H*_*i,CD*_/*H*_*i-1,CD*_	*H*_*i,CD*_ ± σ	*H*_*i,CD*_/*H*_*i-1,CD*_
1	0.004 ± 0.002		0.0001 ± 0.0002		0.02 ± 0.03	–	0.01 ± 0.04		0.001 ± 0.0003	
2	0.01 ± 0.003	2.0	0.0004 ± 0.0004	2.6	0.05 ± 0.06	2.0	0.02 ± 0.07	2.0	0.001 ± 0.0006	2.0
5	0.02 ± 0.01	2.5	0.002 ± 0.001	4.2	0.11 ± 0.13	2.5	0.04 ± 0.17	2.5	0.003 ± 0.002	2.6
10	0.04 ± 0.02	2.0	0.01 ± 0.003	3.3	0.22 ± 0.26	2.0	0.08 ± 0.33	2.0	0.01 ± 0.003	2.1
20	0.08 ± 0.03	2.0	0.02 ± 0.01	3.6	0.43 ± 0.48	1.9	0.17 ± 0.59	2.0	0.01 ± 0.006	2.2
40	0.15 ± 0.06	2.0	0.07 ± 0.02	3.8	0.78 ± 0.8	1.8	0.34 ± 0.94	2.0	0.03 ± 0.01	2.4
60	0.23 ± 0.09	1.5	0.15 ± 0.05	2.2	1.04 ± 0.95	1.3	0.53 ± 1.06	1.5	0.05 ± 0.02	1.7
80	0.30 ± 0.13	1.3	0.26 ± 0.08	1.8	1.23 ± 0.95	1.2	0.72 ± 0.93	1.4	0.08 ± 0.02	1.5
100	0.37 ± 0.016	1.2	0.41 ± 0.12	1.6	1.34 ± 0.79	1.1	0.92 ± 0.56	1.3	0.11 ± 0.03	1.4
>1	1		1		1		1		1	

The *H*_*i*_ values derived for the 2 different dustiness test methods were clearly not similar and different results and correlations would be observed depending on the choice of mean, median, or Q3 values. Significant differences should also be expected if a laboratory has a high variability of dustiness data due to, e.g. particle losses in the setup or less control over the environmental conditions while testing or weighing. Recently, an intra- and inter-laboratory comparison of dustiness methods showed that laboratories coefficient of variation for both methods (SRD and CD) is generally under 20% (Ribalta et al. 2023). However, this coefficient was also observed to reach 40% or more in some specific cases.

**Figure 5.**
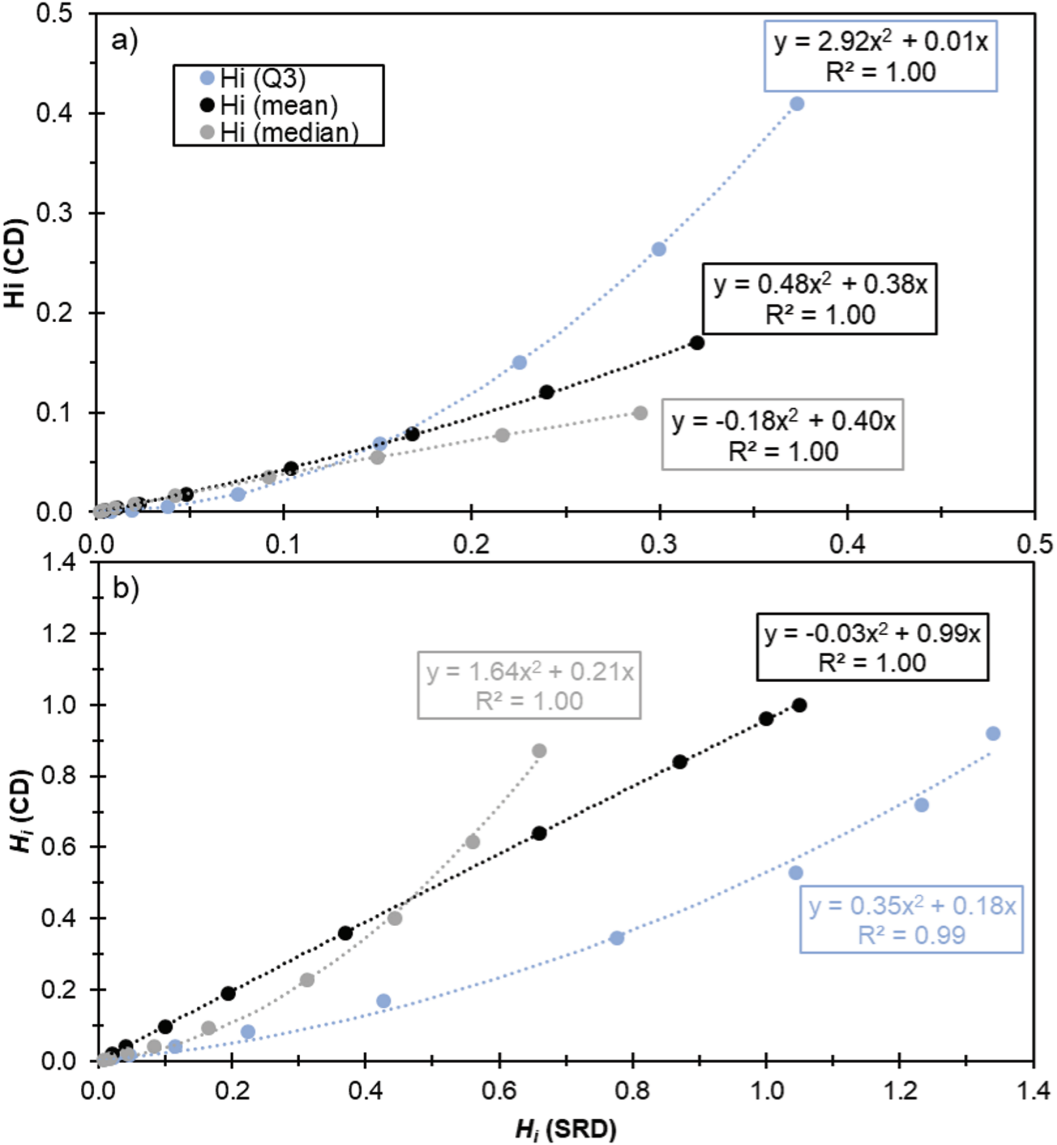
Best fit quadratic correlation equations among the mean, median, and 3^rd^ quartile (Q3) *H*_*i*_ values derived for SRD and CD dustiness testing and based on (a) respirable dustiness mass fraction and (b) number based dustiness index. All equations were forced through (0.0).

Figure 5 shows the relationships between the mean, median, and Q3 *H*_*i*_ values derived for the CD method versus the *H*_*i*_ values derived for the SRD dustiness method. The results show the increasing scatter and difference between *H*_*i*_ values derived for the 2 test methods at increasing both *H*_*i*_ and pouring height. Except for the number-based *H*_*i*_ values derived for SRD (from 60 to 100 cm drop height), all the *H*_*i*_ values obtained for both SRD and CD were <1 meaning that the dustiness tests involved more energy input than the simulated pouring activity and consequently, de-agglomeration and dust generation was higher. This effect was most pronounced in CD dustiness method, especially when analyzing the inhalable and respirable dustiness mass fractions. In addition, all the *H*_*i*_ obtained for the CD were consistently lower than SRD method. Although SRD process shares some similarities with CD dustiness test method, as amounts of powder are raised to a certain height and fall back down, these results are somewhat unexpected. It is anticipated from a mechanistic point of view that the dustiness data generated by the CD test resembles more closely powder pouring than the SRD test method. A possible explanation for these discrepancies is the difference in aerosolization energy used in both systems. In both CD and SRD dustiness methods, the particles in the bulk sample can be assumed to be subjected to (i) the vertical gravitational force, (ii) the drag force acting as a separation force; and (iii) surface forces between the particles binding them together. In CD test method, a drag force is exerted on the dropped material in upwards direction (opposed to the material fall direction). This drag force was observed to be one of the main drivers of powder aerosolization during CD test (Shandilya et al. 2019). The presence of this drag force likely generates resuspension of respirable and coarser particles, which would not occur during a pouring process as the one resembled in the experiments presented here. It is probable that the agglomerated particles break up into smaller particles, when subjected to dispersion forces such as the ones used in CD method (Ding et al. 2017). In general, the interplay of de-agglomeration mechanism justifies the lowest *H*_*i*_ values obtained, especially for the CD test method. On the other hand, it is also plausible that the gravitational force exerted in the SRD is relatively higher than the drag force acting on the particles and therefore less tendency to emit airborne particles and subsequently lower dustiness indexes. Further tests and deeper analysis of the dustiness data is likely needed to validate and/or improve our understanding of these observations. Moreover, additional studies are needed to derive new *H*_*i*_ and improve exposure modelling performance based on dustiness testing (e.g. expanding to other materials, and handling exposure scenarios). The study from Ribalta *et al.*, (2024), demonstrated the applicability and usability of the dustiness indexes for emission source characterization and subsequent exposure modelling of powder handling scenarios by using the *H*_*i*_ factors derived from this study. The source term emission rates determined by using Equation 1 were applied as input parameter in a tailored 2-box mass balance model (also known as near-field (NF)/far-field (FF) model; Ganser and Hewett, 2017) to calculate estimated exposure concentrations of several exposure scenarios which were compared to measured exposure concentrations afterwards. Overall, these results showed a clear improvement in the modelling output when using *H*_*i*_ (ratio of modelled/measured concentrations ranging from 0.9 to 10 in 75% of cases versus 17% of the cases when not using *H*_*i*_). This supports the strategy of applying derived *H*_*i*_ values in exposure assessment tools such as in GUIDEnano and NanoSafer, which use the dustiness data as input for emissions source characterization.

## Conclusions

To move forward in human exposure assessment, there is an increasing need to use strong and validated predictive models for exposure assessment to both nanomaterials and non-nanomaterials to meet the recent increased focus on general risk assessment and management. Human exposure modelling usually requires quantitative process-specific release data and emission characteristics in the potential exposure scenario. This study provides valuable new information deriving experimental handling energy factors (*H*_*i*_) for conversion of dustiness data to emission rates for powder handling activities in exposure assessment modelling considering both the number- and mass-based respirable, and inhalable dust release fraction as determined by the EN17199-3 CD and the EN17199-4 SRD test methods.

The results showed increasing scatter and difference between *H*_*i*_ values derived for the two test methods at increasing *H*_*i*_ and pouring height. For precautionary reasons, the determined *H*_*i*_ values were based on the upper 3^rd^ quartile of the individual calculated *H*_*i*_. Nearly all the *H*_*i*_ values obtained for both SRD and CD were <1, concluding that the mechanical energy applied in the simulated pouring processes at heights <1 m is lower than the energy applied in the dustiness test methods. The effective dust generation and dispersion effect was most pronounced in CD dustiness test method, especially when analyzing the mass-based dustiness. This overall result indicates that the SRD mechanistically resembles more closely the powder studied pouring activity. The energy input and the de-agglomeration mechanism may be the responsible driver for the generation of respirable aerosols. However, further studies on additional powders are needed to improve our understanding and validate this hypothesis. Despite limitations in coverage of different powder use and handling scenarios, this work presents the first known attempt to improve exposure modelling based on dustiness testing.

## Supplementary Material

wxae009_suppl_Supplementary_Material

## Data Availability

Dustiness data are available in the caLIBRAte data entity accessible from the eNANOMAPPER data infrastructure. The other data presented in this study are available on request from the corresponding author.
