# Supplementary material for: Development of handling energy factors for use of dustiness data in exposure assessment modelling
Source: Ann Work Expo Health. 2024 Feb 24;68(3):295–311. doi: 10.1093/annweh/wxae009 (PMC10941727; doi:10.1093/annweh/wxae009)
Supplement: wxae009_suppl_Supplementary_Material [file wxae009_suppl_supplementary_material.pdf]

Supplementary material

Development of handling energy factors for use of dustiness data in exposure assessment modelling

Ana Sofia Fonseca [1,*], Carla Ribalta [1,2], Neeraj Shandilya [3], Wouter Fransman [3], Keld Alstrup Jensen [1]

[1] National Research Centre for the Working Environment (NRCWE), Lersø Parkallé 105, DK-2100 Copenhagen, Denmark; agf@nfa.dk (A.S.F.); ribaltacarrasco.carla@baua.bund.de (C.R.); kaj@nfa.dk (K.A.J.)

[2] Federal Institute for Occupational Safety and Health (BAuA), 10317 Berlin, Germany; ribaltacarrasco.carla@baua.bund.de (C.R.)

[3] TNO, Risk Assessment of Products In Development, Utrechtseweg 48, 3704 HE Zeist, Netherlands; neeraj.shandilya@tno.nl (N.S.); wouter.fransman@tno.nl (W.F.)

*Correspondence: agf@nfa.dk (A.S.F.); Phone: +45 20 59 45 05

Annex I. Evaluation of dustiness parameters

The respirable dustiness mass fraction (in mg kg^{-1}) from SRD was determined according to the equation below, following the procedures given in EN 17199-4:2019:

$$DI_{m-SRD}\left[\frac{\text{mg}}{\text{kg}}\right] = \frac{Q_A \times m_f}{Q_{B1} \times m}$$

where Q_A and Q_{B1} are the air volume flows through the SRD and cyclone, respectively (in L min^{-1}), m_f is the blank-filter corrected mass of dust collected on the cyclone filter (in mg) and m is the mass of powder used in the test in kg.

For CD, the respirable (DI_{m-CD}) and inhalable $DI_{m,inhalable-CD}$ dustiness mass fractions were determined according to the following equations (EN 15051-3: 2013):

$$DI_{m-CD} = \frac{\Delta m_R}{m_c} . \frac{Q_{tot}}{Q_R} \qquad \text{and} \qquad DI_{m,inhalable-CD} = \frac{\Delta m_I}{m_c} . \frac{Q_{tot}}{Q_I}$$

where Δm_R is the mass of the dust collected by the sampler for respirable dust (in mg), Δm_I is the mass of the dust collected by the sampler for inhalable dust (in mg), m_c is the drop mass in the collector tank (in kg), Q_R is the flow rate of the sampler for respirable dust (in L min^{-1}), Q_I is the flow rate of the sampler for inhalable dust (in L min^{-1}), Q_{tot} is the total flow rate (in L min^{-1}) and $Q_{tot} = Q_R + Q_I + Q_m$ (Q_m is the main pump flow rate (in L min^{-1}).

Additionally, the number-based dustiness index DI_N (mg^{-1}) for SRD, was calculated accordingly:

$$DI_{N-SRD} = \frac{\overline{S_{SRD}}}{md} \cdot 60s$$

Where *md* (in mg), is the powder mass loaded into the dustiness system, and $\overline{S_{SRD}}$ is the average number-based emission rate during the 60 s rotation (s^{-1}) determined by Eq.8.

DI_N for CD was calculated according to the following equation (EN 17199-3:2019):

$$DI_{N-CD} = \frac{\bar{C}_{N,t} \times \dot{V} \times t_t}{m_0}$$

where $\bar{C}_{N,t}$ is the average number concentration measured by the CPC (in cm^{-3}), $\dot{V}$ is the volume air flow rate of the CD (53 000 cm^3 min^{-1}), t_t is the whole test duration (9 min) and m_0 is the mass fed during the whole test duration (in mg).

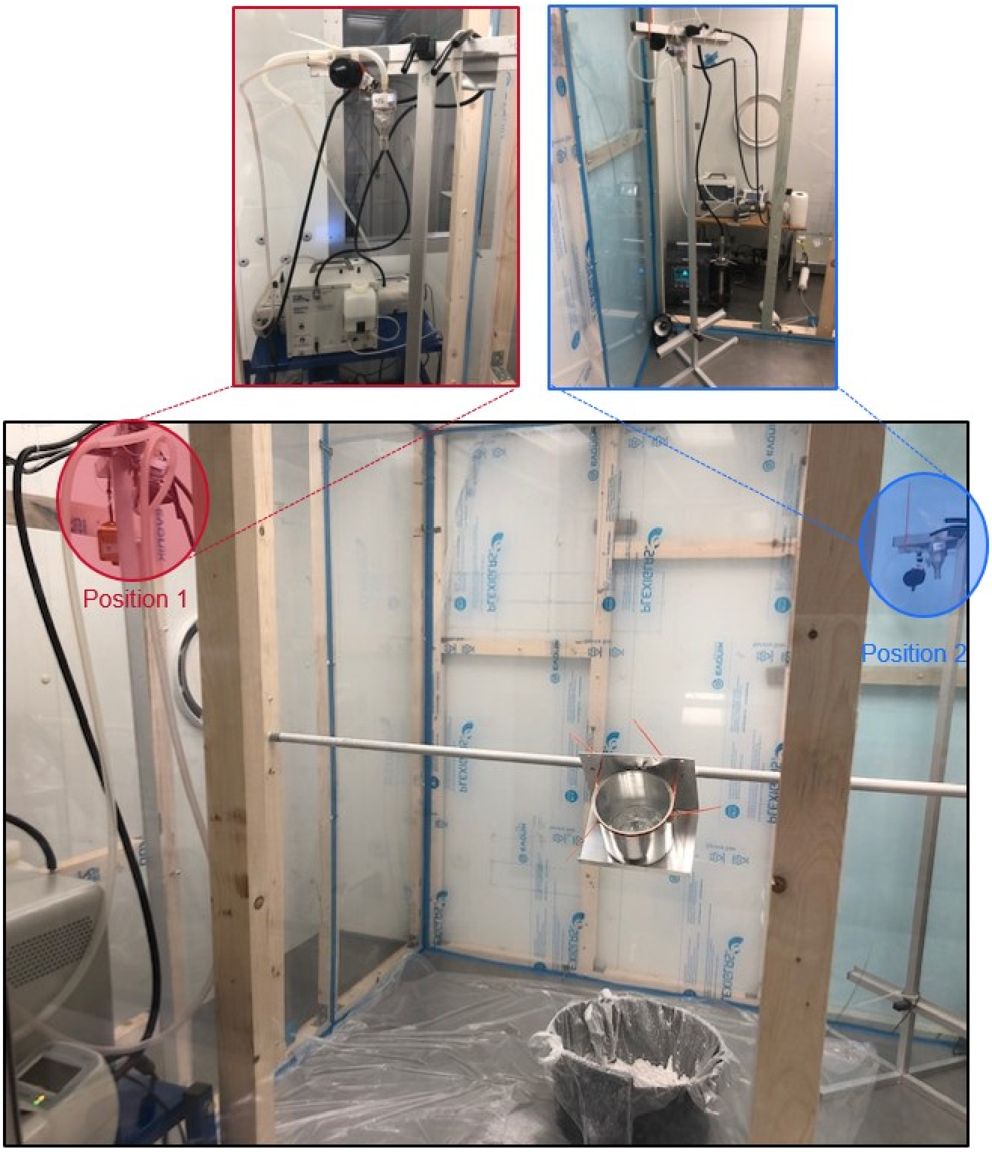

Figure S1. Pictures of the test chamber and sampling positions.

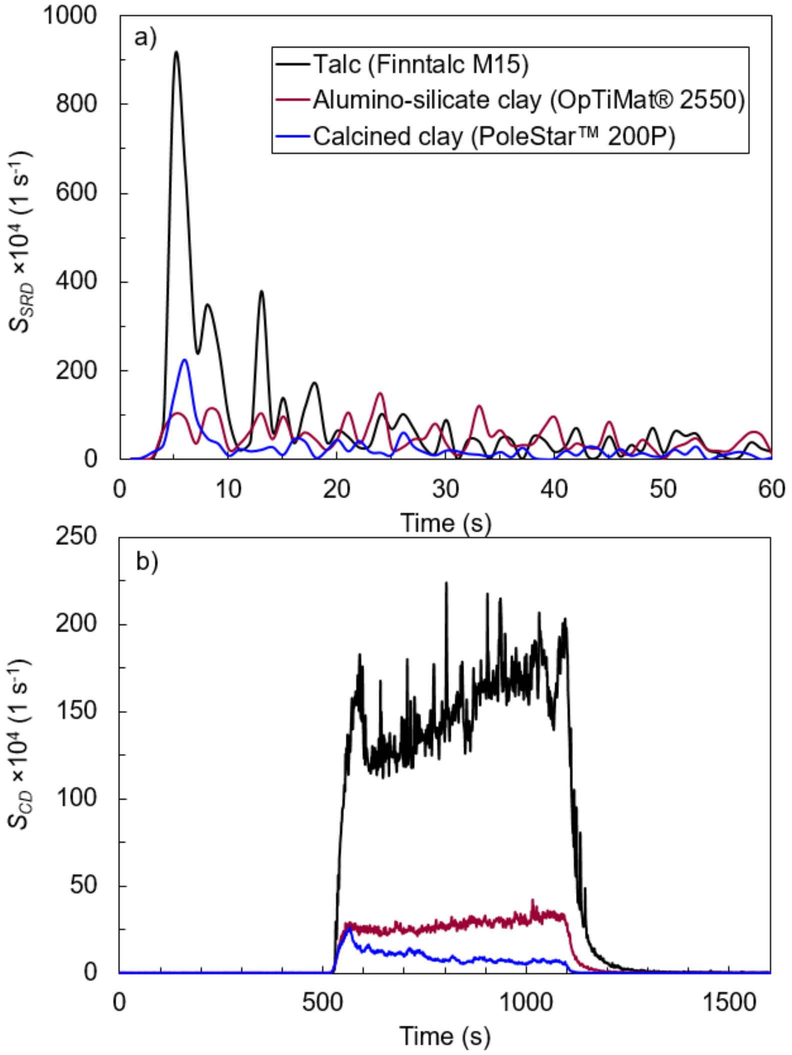

Figure S2. Number-based average emission rates based on CPC data obtained by a) the SRD dustiness method, and b) the CD dustiness method.

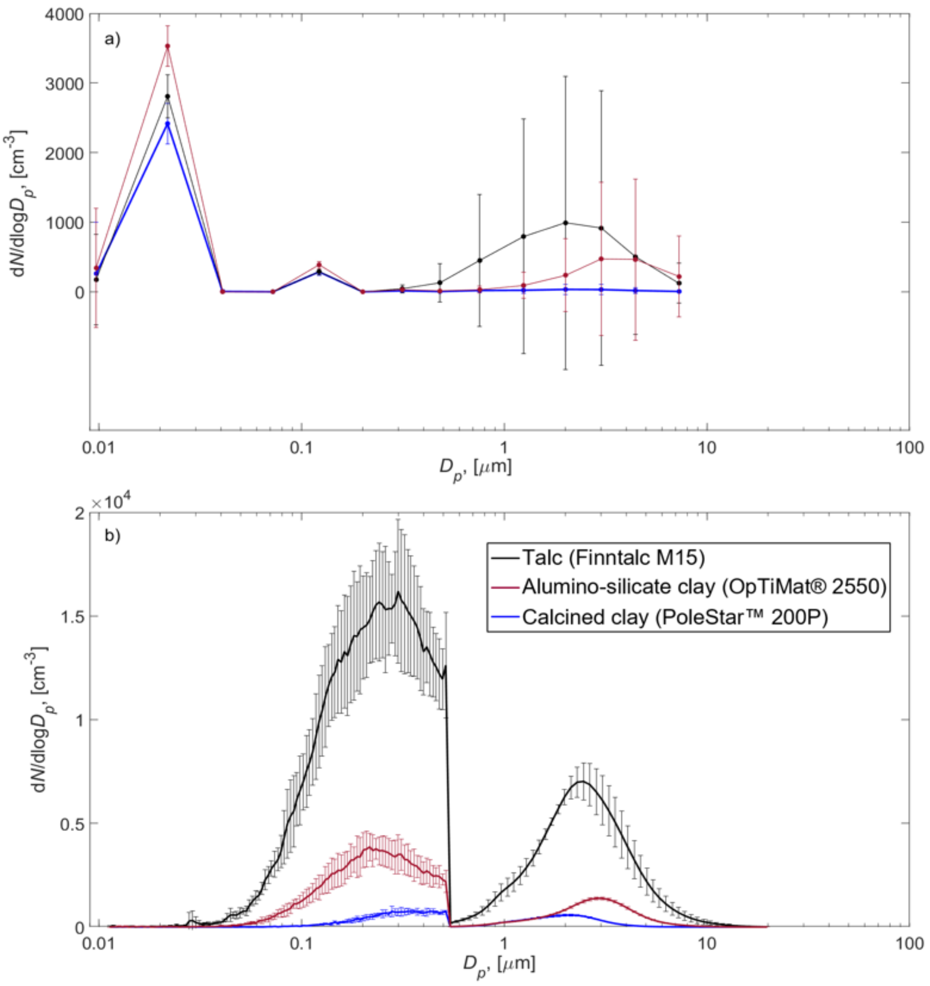

Figure S3. Mean particle number size distributions in a) the SRD dustiness method by ELPI, and b) the CD method by SMPS and APS, TSI. Error bars show the standard deviation.

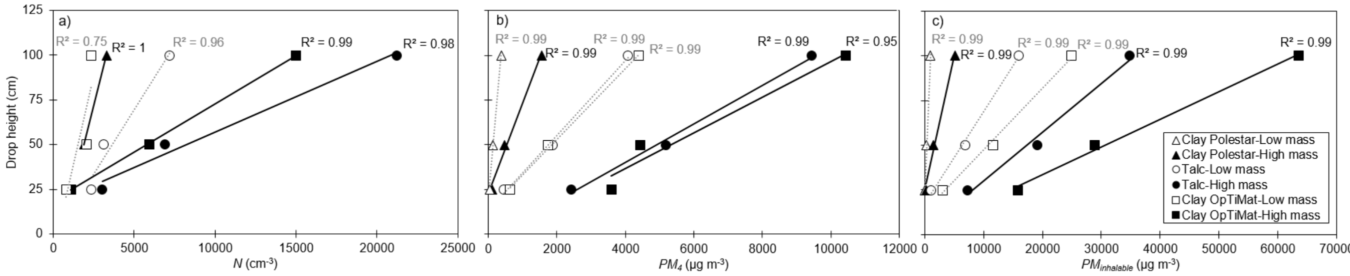

Figure S4. Averages of particle concentrations measured during the drop tests as a function of drop height: a) maximum of particle number concentration; b) mean respirable particle mass concentration; c) mean inhalable particle mass concentration.

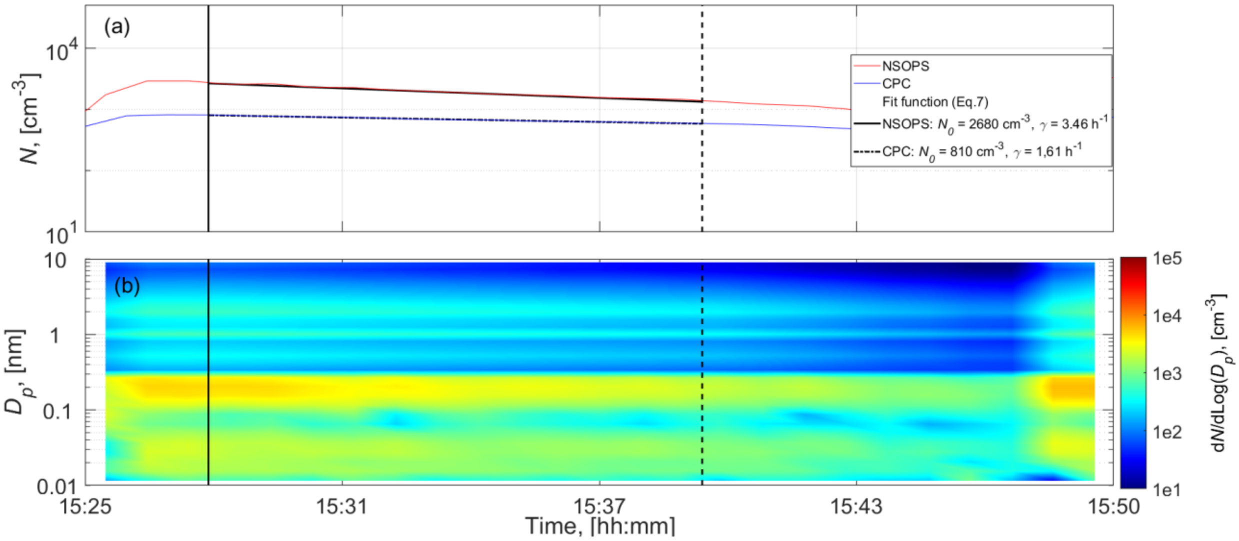

Figure S5. Characterization of particles decay parameter (γ in h^{-1}) for clay PoleStar material. Figure a) shows the exponential decay function fitted to the particle number concentrations measured by the NSOPS and CPC in position 1 and b) shows particle size distributions measured by the NSOPS (position 1). Solid and dashed vertical black lines show the start and end time used to derive the decay rates.

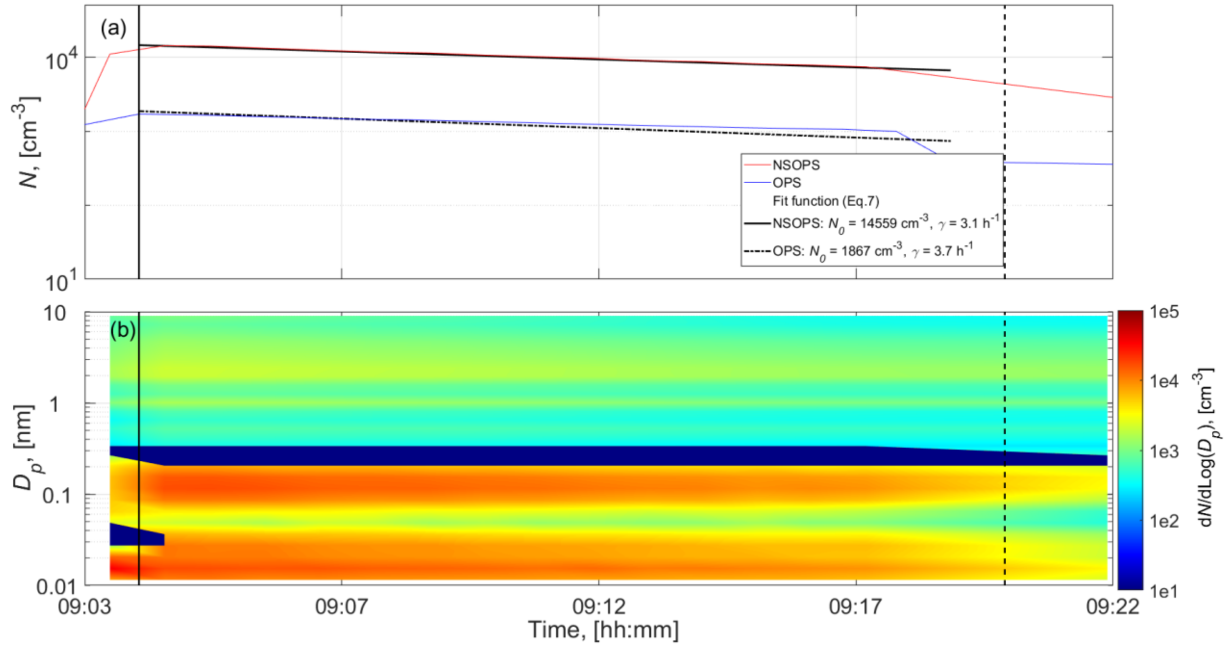

Figure S6. Characterization of particles decay parameter (γ in h^{-1}) for talc material. Figure a) shows the exponential decay function fitted to the particle number concentrations measured by the NSOPS and OPS in position 1 and b) shows particle size distributions measured by the NSOPS (position 1). Solid and dashed vertical black lines show the start and end time used to derive the decay rates.

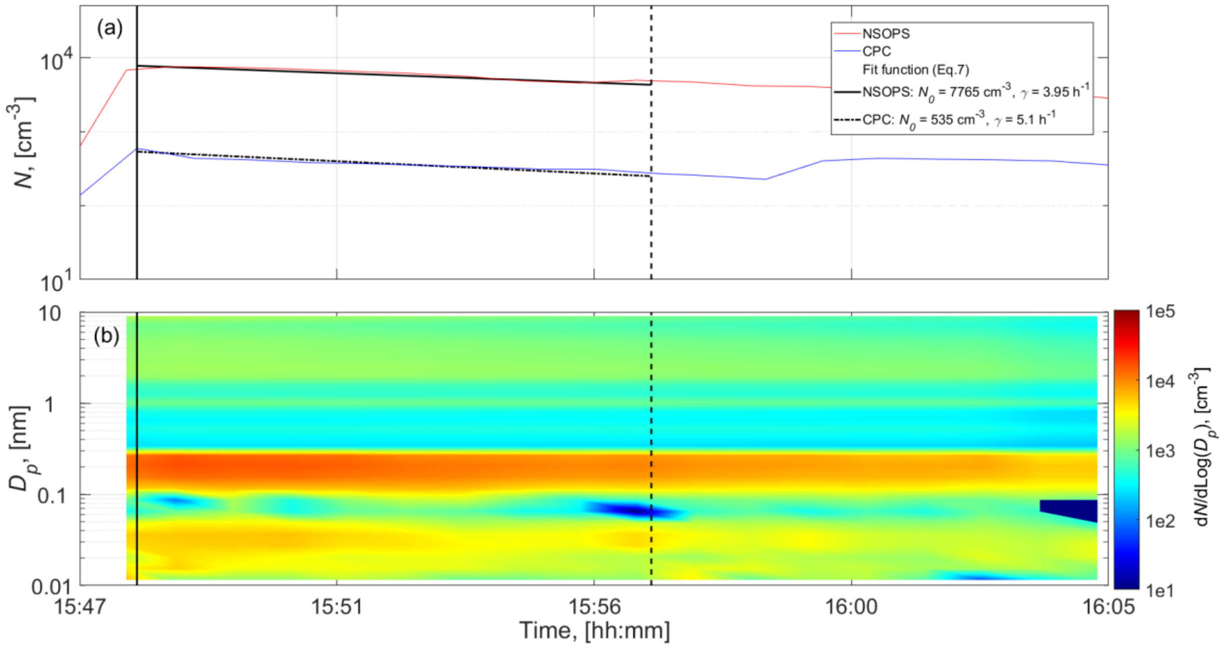

Figure S7. Characterization of particles decay parameter (γ in h^{-1}) for clay OpTiMat material. Figure a) shows the exponential decay function fitted to the particle number concentrations measured by the NSOPS and CPC in position 1 and b) shows particle size distributions measured by the NSOPS (position 1). Solid and dashed vertical black lines show the start and end time used to derive the decay rates.

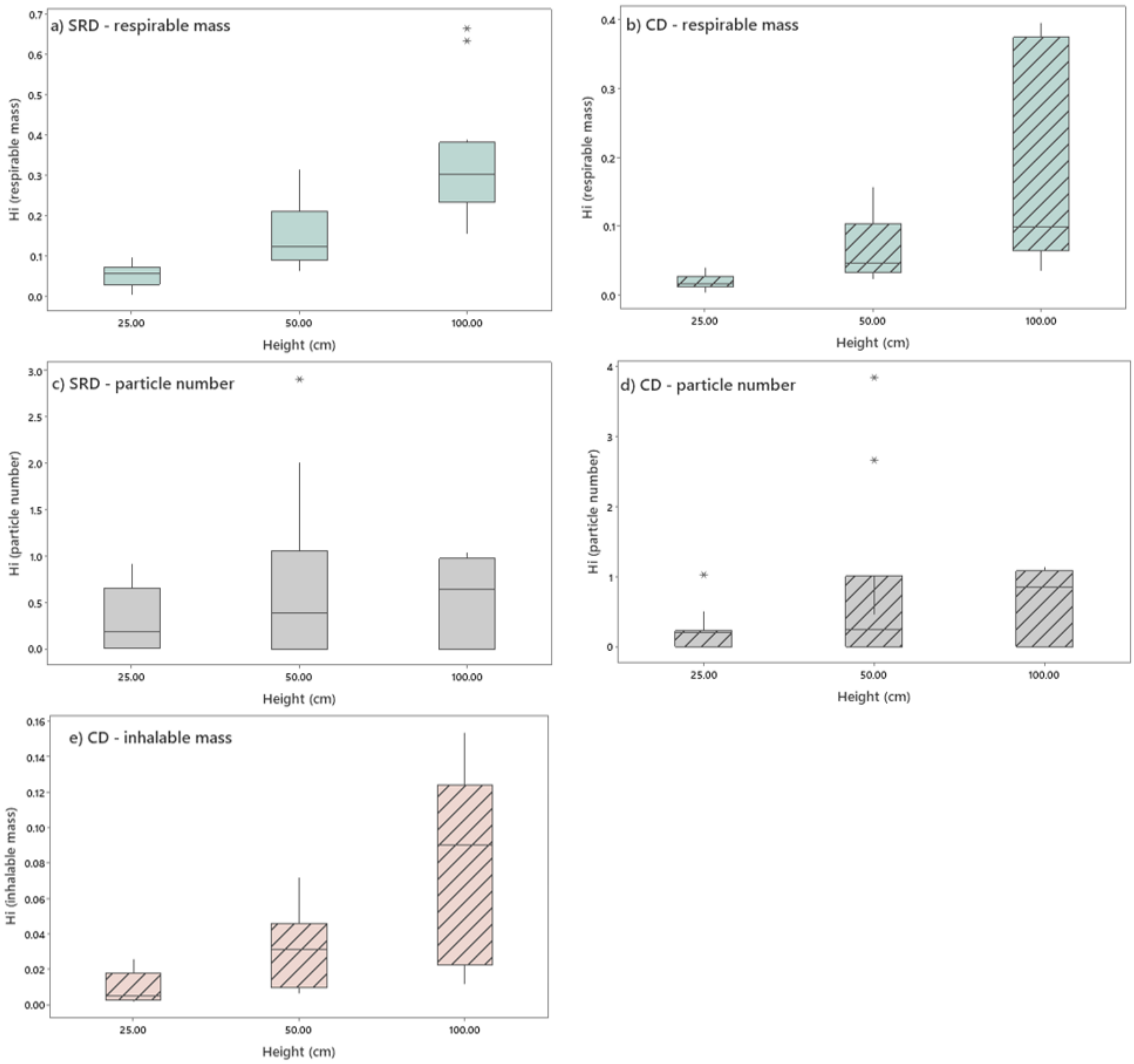

Figure S8. First quartile (Q1), median, and third quartile (Q3) box plots of H_i values calculated for respirable and inhalable particle mass and number concentrations for both SRD and CD methods for each of the pouring heights. Bars show the complete data range for each pouring height and symbols * show the outliers.

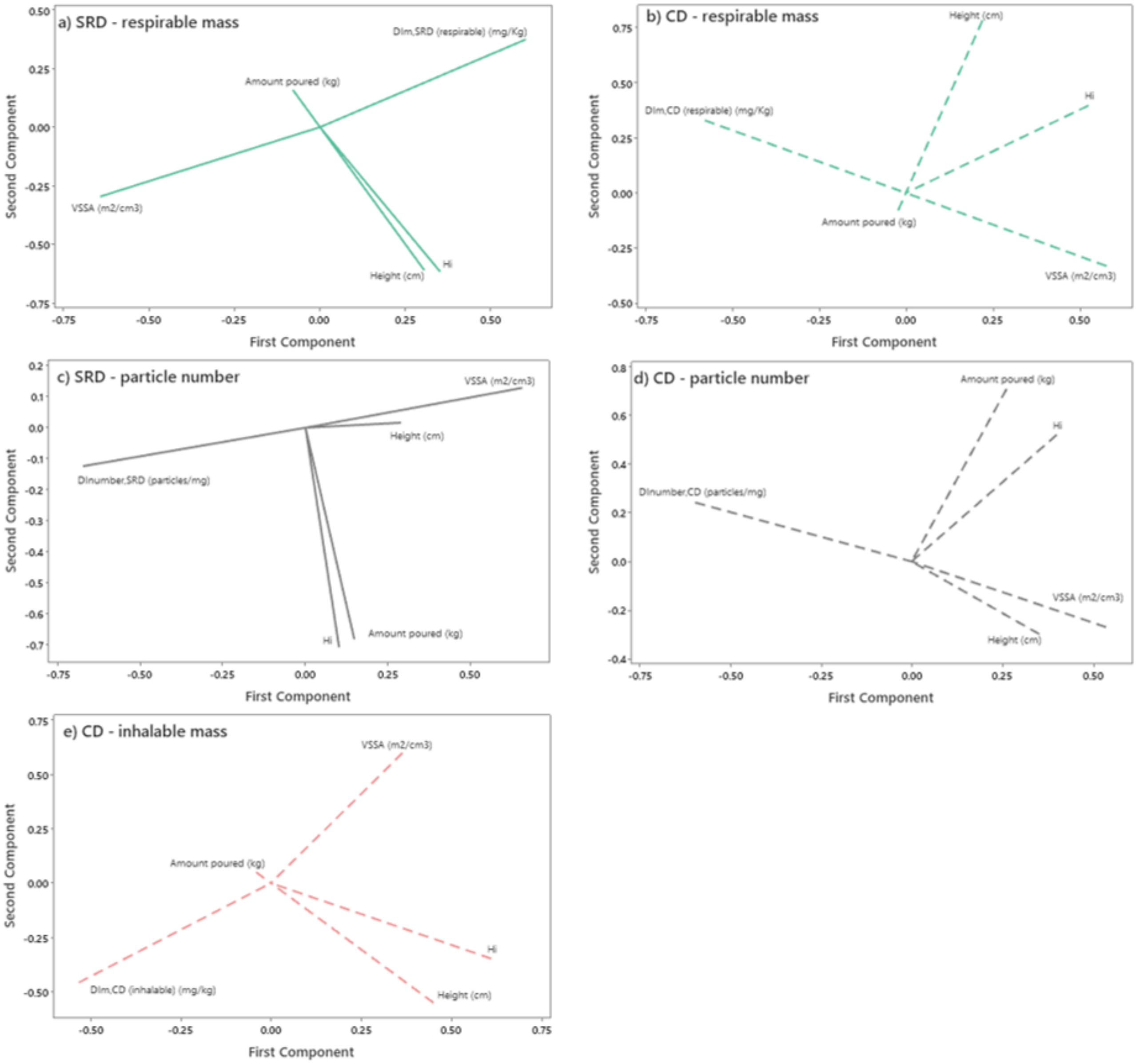

Figure S9. Loading plot for principal component analysis of parameters potentially correlating with H_i calculated for particle mass and number concentrations for both SRD and CD methods.

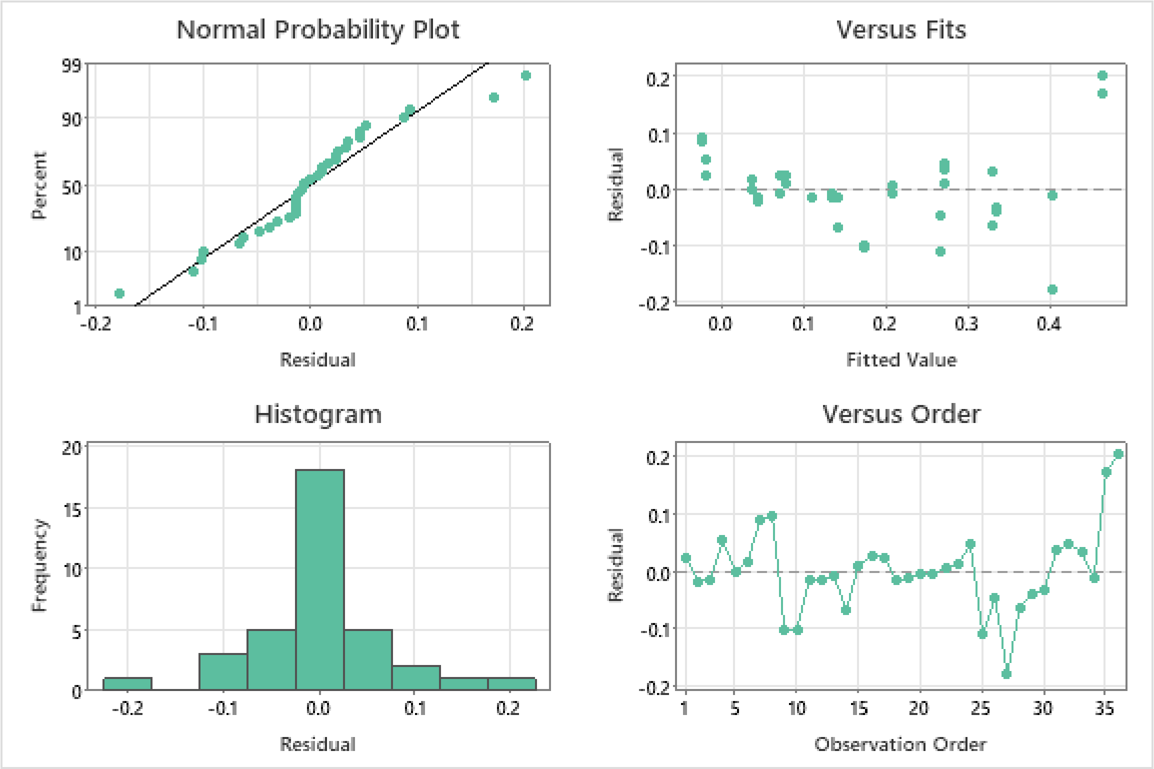

Figure S10. Residual plots for prediction of all H_i values (determined by respirable dustiness mass fraction) for the SRD dustiness method based on the drop height, respirable dustiness mass fractions, VSSA, and amount of powder poured.

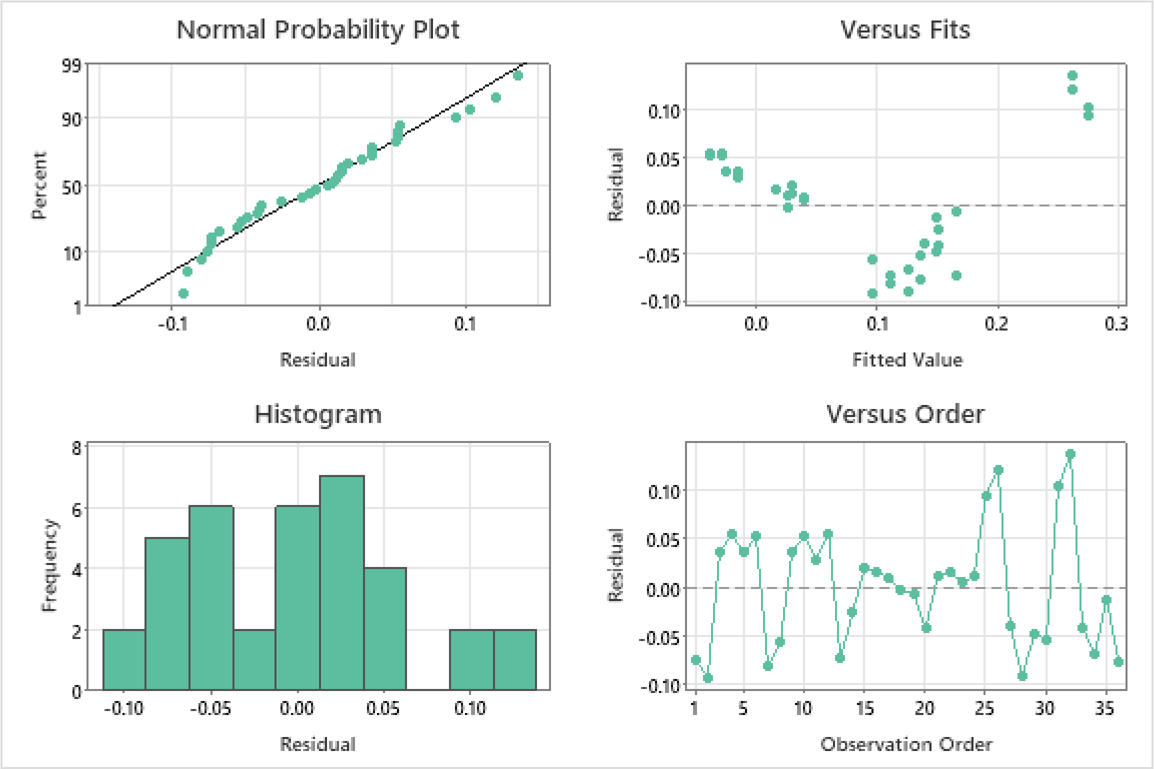

Figure S11. Residual plots for prediction of all H_i values (determined by respirable dustiness mass fraction) for the CD dustiness method based on the drop height, respirable dustiness mass fractions, VSSA, and amount of powder poured.

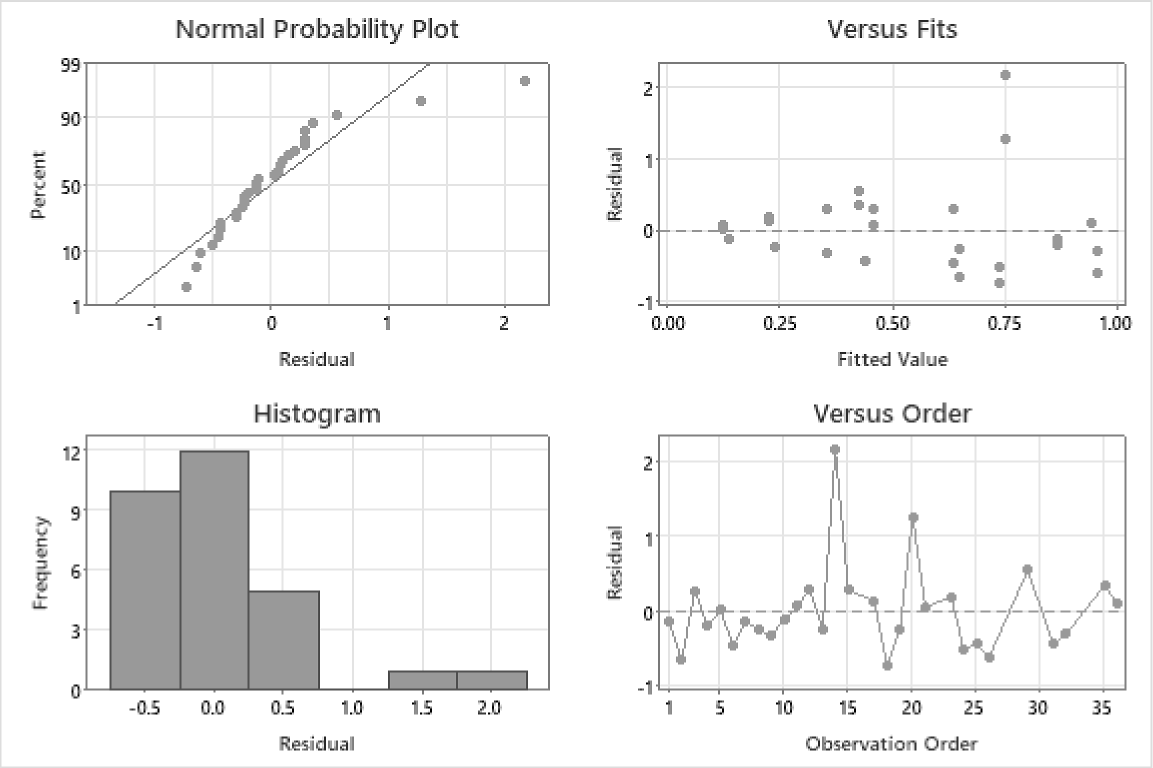

Figure S12. Residual plots for prediction of all H_i values (determined by number based dustiness index) for the SRD dustiness method based on the drop height, number based dustiness index, VSSA, and amount of powder poured.

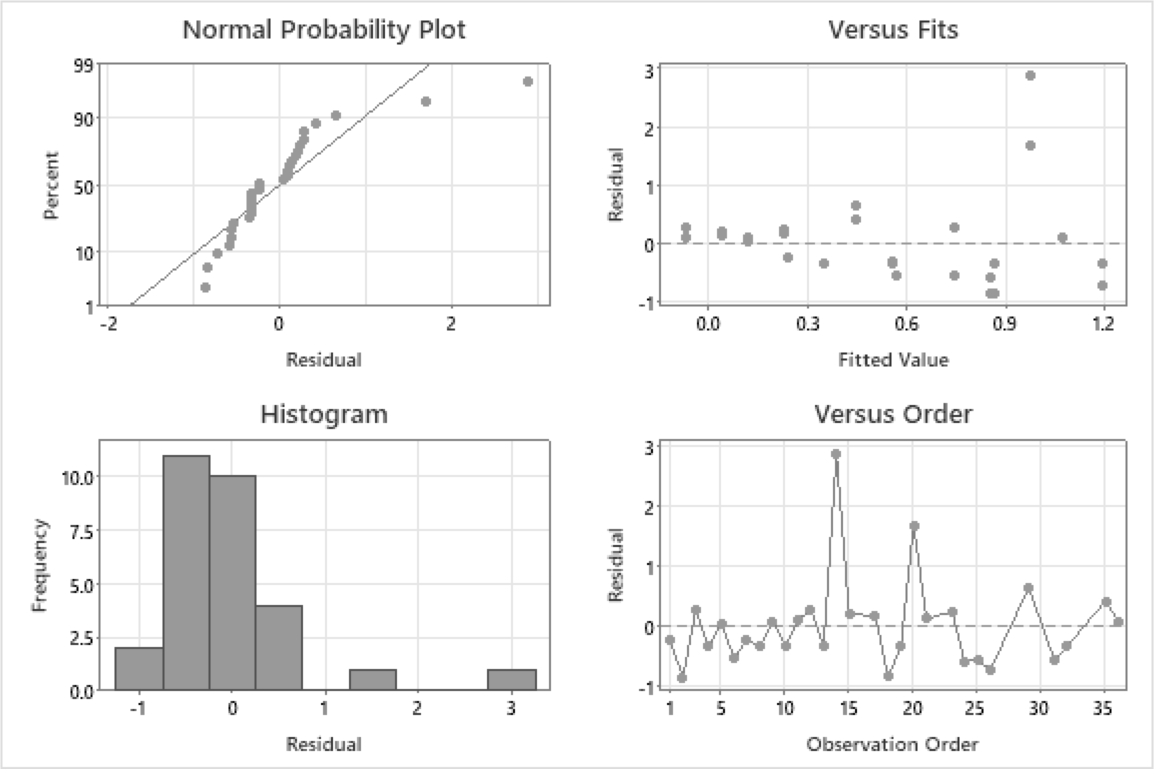

Figure S13. Residual plots for prediction of all H_i values (determined by number based dustiness index) for the CD dustiness method based on the drop height, number based dustiness index, VSSA, and amount of powder poured.

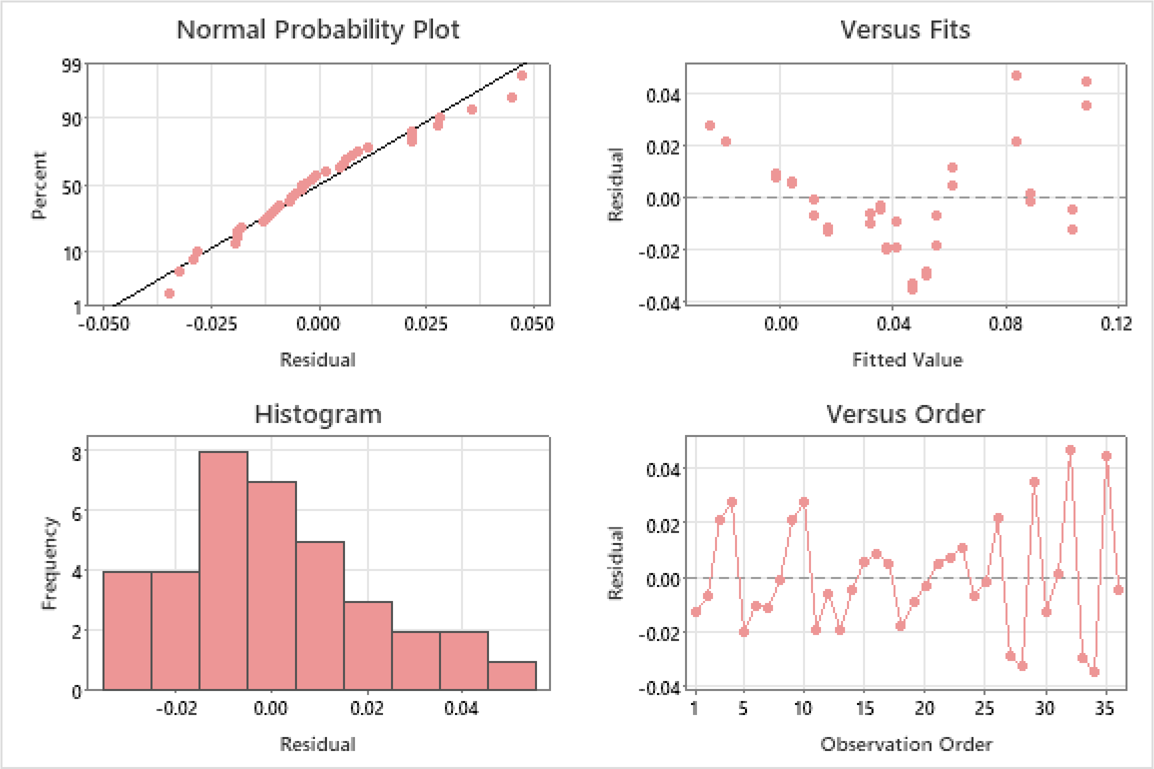

Figure S14. Residual plots for prediction of all H_i values (determined by inhalable dustiness mass fraction) for the CD dustiness method based on the drop height, inhalable dustiness mass fractions, VSSA, and amount of powder poured.

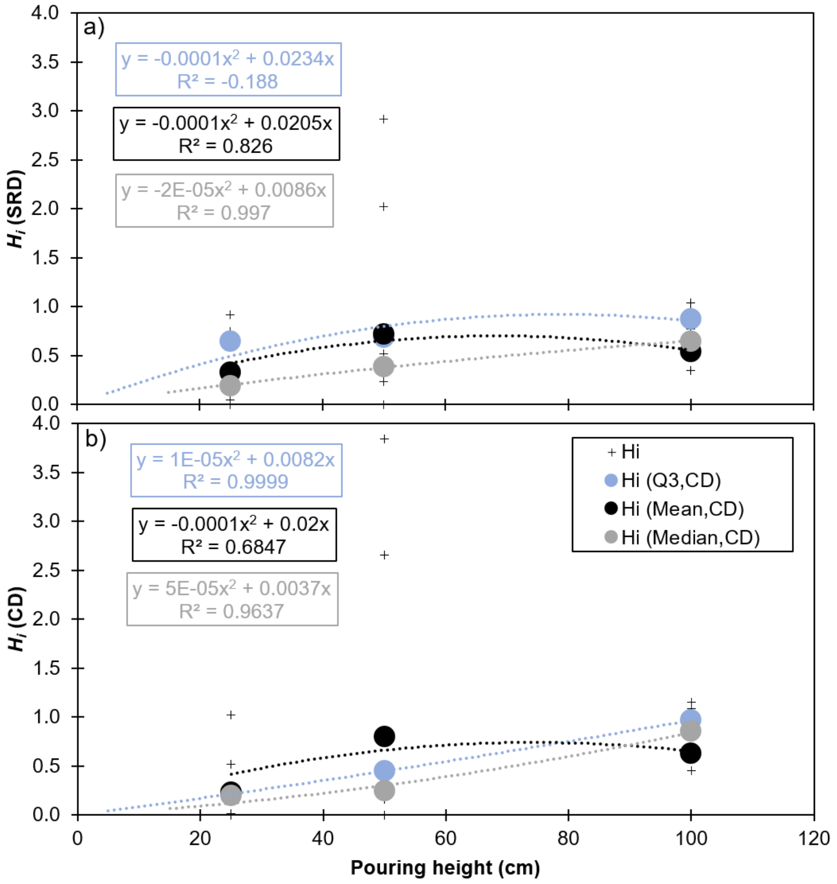

Figure S15. Individual calculated H_i values (determined by number based dustiness index), regression curves and functions for the mean, median, and upper 3rd quartile (Q3) values for SRD (a) and CD dustiness method (b).

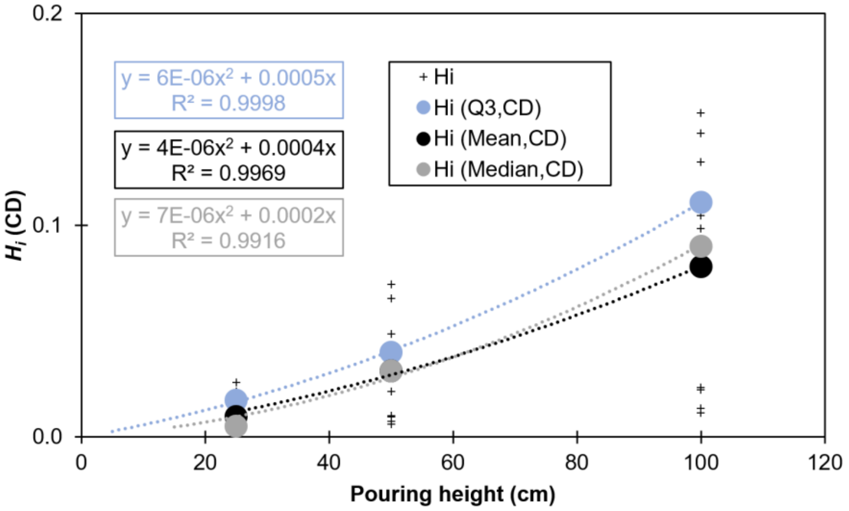

Figure S16. Individual calculated H_i values (determined by inhalable dustiness mass fraction), regression curves and functions for the mean, median, and upper 3rd quartile (Q3) values for CD dustiness method.

Table S1. Particle concentrations obtained during drop tests. BG: background (5 min before the drop tests); *N*: particle number concentration; PM_4: respirable mass concentration obtained by cumulative gravimetric sampling; GM: geometric mean; GSD: geometric standard deviation; GMD: geometric mean diameter measured by NSOPS; N/A: Not available data. The handling energy factors marked grey resulted from the gravimetrical analysis of the filters collected during the drop tests which might have higher uncertainties.

		Amount poured (kg)	Drop height (cm)	Sampling duration (min)	$N_{NSOPS, pos1}$ (cm^{-3})		$N_{CPC, pos1}$ (cm^{-3})		$N_{CPC, pos2}$ (cm^{-3})		$PM_{4\ pos1}$ (µg m^{-3})	$PM_{4\ pos2}$ (µg m^{-3})		Respirable mass D_p (measured by NSOPS; nm)		$PM_{inhalable\ pos1}$ (µg m^{-3})	$PM_{inhalable\ pos2}$ (µg m^{-3})	Inhalable mass D_p (measured by NSOPS; nm)	
					GM	GSD	GM	GSD	GM	GSD				GMD	GSD			GMD	GSD
**Exp1**	BG	0.75 (3 × 0.25 kg)	25	33	N/A	N/A	1201	1	983	1	N/A	N/A	N/A	N/A	N/A	N/A	N/A	N/A	N/A
**(clay Polestar)**	Pouring				N/A	N/A	1163	1	916	1	36.2[a]	28.7[a]		3120	1.7	46.5	63.2	5342	1.7
**Exp2**	BG	3 (3 × 1 kg)	25	13	N/A	N/A	848	1	701	1	N/A	N/A	N/A	N/A	N/A	N/A	N/A	N/A	N/A
**(clay Polestar)**	Pouring				N/A	N/A	827	1	684	1	15.1[a]	106.1[a]		3263	1.6	199.2	318.3	5452	1.7
**Exp3**	BG	0.75 (3 × 0.25 kg)	50	16	N/A	N/A	818	1	733	1	N/A	N/A	N/A	N/A	N/A	N/A	N/A	N/A	N/A
**(clay Polestar)**	Pouring				N/A	N/A	796	1	701	1	91.1	156.9		3133	1.6	237	346	5084	1.7
**Exp4**	BG	3 (3 × 1 kg)	50	15	303	1.0	209	1	176	1	N/A	N/A	N/A	N/A	N/A	N/A	N/A	N/A	N/A
**(clay Polestar)**	Pouring				797	3.4	282	1	226	1	500.3	433.2		413	4.2	1362	1411	2076	5.7
**Exp5**	BG	0.75 (3 × 0.25 kg)	100	17	231	NaN	216	1	167	1	N/A	N/A	N/A	N/A	N/A	N/A	N/A	N/A	N/A
**(clay Polestar)**	Pouring				490	2.7	224	1	198	1	366.9	376.3		493	4.2	948	982	2205	5.3
**Exp6**	BG	3 (3 × 1 kg)	100	16	744	1.2	401	1.0	264	1.1	N/A	N/A	N/A	N/A	N/A	N/A	N/A	N/A	N/A
**(clay Polestar)**	Pouring				1897	2.5	505	1.4	340	1.5	1521	1581		417	3.9	4577	5678	1685	5.7
**Exp7**	BG	0.75 (3 × 0.25 kg)	25	31	226	1.0	212	1.1	181	1.1	N/A	N/A	N/A	N/A	N/A	N/A	N/A	N/A	N/A
**(Talc)**	Pouring				764	4.9	261	1.5	188	1.1	459.5	441.9		1071	5.2	1136	1094	4500	3.4
**Exp8**	BG	3 (3 × 1 kg)	25	16	84	1.1	99	1.1	75	1.1	N/A	N/A	N/A	N/A	N/A	N/A	N/A	N/A	N/A
**(Talc)**	Pouring				861	6.0	149	1.6	128	1.5	2480	2343		531	4.6	7729	6700	3063	4.7
**Exp9**	BG	0.75 (3 × 0.25 kg)	50	16	463	1.1	495	1.0	360	1.0	N/A	N/A	N/A	N/A	N/A	N/A	N/A	N/A	N/A
**(Talc)**	Pouring				1635	2.5	556	1.2	408	1.1	2040	1709		822	5.1	7131	6648	3989	3.8
**Exp10**	BG	3 (3 × 1 kg)	50	16	294	1.1	N/A	N/A	N/A	N/A	N/A	N/A	N/A	N/A	N/A	N/A	N/A	N/A	N/A
**(Talc)**	Pouring				2374	5.7	N/A	N/A	N/A	N/A	5185	5185		887	5.3	21164	17054[b]	4271	3.6
**Exp11**	BG	0.75 (3 × 0.25 kg)	100	16	865	1.1	N/A	N/A	N/A	N/A	N/A	N/A	N/A	N/A	N/A	N/A	N/A	N/A	N/A
**(Talc)**	Pouring				3505	3.2	N/A	N/A	N/A	N/A	4104	4048		885	5.2	16295	15562	4221	3.6
**Exp12**	BG	3 (3 × 1 kg)	100	17	840	1.1	N/A	N/A	N/A	N/A	N/A	N/A	N/A	N/A	N/A	N/A	N/A	N/A	N/A
**(Talc)**	Pouring				18084	1.3	N/A	N/A	N/A	N/A	5750[b]	9446		865	5.4	37922	31764	4316	3.7
**Exp13**	BG	0.75 (3 × 0.25 kg)	25	31	152	1.6	106	1.1	108	1.1	N/A	N/A	N/A	N/A	N/A	N/A	N/A	N/A	N/A
**(clay OpTiMat)**	Pouring				749	1.1	115	1.2	116	1.2	750.1	511.1		557	5.2	2908	3015	4111	4.1
**Exp14**	BG	3 (3 × 1 kg)	25	16	109	1.0	61	1.1	64	1.1	N/A	N/A	N/A	N/A	N/A	N/A	N/A	N/A	N/A
**(clay OpTiMat)**	Pouring				445	3.6	67	1.4	96	1.9	3388	3784		676	5.1	14432	17289	4277	3.8
**Exp15**	BG	0.75 (3 × 0.25 kg)	50	16	161	1.0	117	1.1	81	1.1	N/A	N/A	N/A	N/A	N/A	N/A	N/A	N/A	N/A
**(clay OpTiMat)**	Pouring				705	4.5	132	1.2	97	1.2	1799 [b]	1681		793	5.3	11003	12097	4796	3.4
**Exp16**	BG	3 (3 × 1 kg)	50	16	359	1.9	N/A	N/A	51	1.1	N/A	N/A	N/A	N/A	N/A	N/A	N/A	N/A	N/A
**(clay OpTiMat)**	Pouring				930	5.4	N/A	N/A	71	1.5	3470	5400		479	4.5	25015	32717	3341	4.7
**Exp17**	BG	0.75 (3 × 0.25 kg)	100	16	106	1.0	94	1.1	70	1.1	N/A	N/A	N/A	N/A	N/A	N/A	N/A	N/A	N/A
**(clay OpTiMat)**	Pouring				1980	1.3	136	1.5	103	1.6	3721	5057		626	5.1	24109	25700	4436	3.8
**Exp18**	BG	3 (3 × 1 kg)	100	17	256	1.5	N/A	N/A	91	1.1	N/A	N/A	N/A	N/A	N/A	N/A	N/A	N/A	N/A
**(clay OpTiMat)**	Pouring				1494	26.1	N/A	N/A	130	1.9	12192 [b]	8679		304	2.9	60867	66184	1627	6.1

a below detection limit

b mass that was probably lost during filter handling

Table S2. Particle number emission rates (*S*) obtained during drop tests. GM: geometric mean; GSD: geometric standard deviation; N/A: Not available data.

		Amount poured (kg)	Drop height (cm)	$S_{CPC, pos1}$ x 10^6 (min^{-1})		$S_{CPC, pos2}$ x 10^6 (min^{-1})		$S_{NSOPS, pos1}$ x 10^6 (min^{-1})	
				GM	GSD	GM	GSD	GM	GSD
**Exp1**	BG	0.75 (3 × 0.25 kg)	25	420.3	1.0	338.4	1.1	N/A	N/A
**(clay Polestar)**	Pouring			385.0	1.0	303.3	1.0	N/A	N/A
**Exp2**	BG	3 (3 × 1 kg)	25	283.7	1.0	223.5	1.1	N/A	N/A
**(clay Polestar)**	Pouring			273.7	1.0	226.4	1.0	N/A	N/A
**Exp3**	BG	0.75 (3 × 0.25 kg)	50	284.4	1.0	254.3	1.0	N/A	N/A
**(clay Polestar)**	Pouring			263.5	1.0	231.9	1.0	N/A	N/A
**Exp4**	BG	3 (3 × 1 kg)	50	71.4	1.1	59.5	1.1	104.2	1.0
**(clay Polestar)**	Pouring			93.3	1.3	74.7	1.2	526.3	1.3
**Exp5**	BG	0.75 (3 × 0.25 kg)	100	105.6	1.7	66.7	2.7	74.9	1.1
**(clay Polestar)**	Pouring			74.1	1.2	65.4	1.3	162.2	2.7
**Exp6**	BG	3 (3 × 1 kg)	100	164.4	1.2	107.7	1.2	337.3	1.2
**(clay Polestar)**	Pouring			167.0	1.4	112.5	1.5	477.1	3.1
**Exp7**	BG	0.75 (3 × 0.25 kg)	25	96.8	1.1	81.7	1.1	104.1	1.1
**(Talc)**	Pouring			115.0	1.5	82.9	1.1	336.7	4.9
**Exp8**	BG	3 (3 × 1 kg)	25	46.5	1.1	34.9	1.1	44.9	1.1
**(Talc)**	Pouring			65.8	1.6	56.4	1.5	202.9	7.5
**Exp9**	BG	0.75 (3 × 0.25 kg)	50	223.7	1.0	164.8	1.1	210.7	1.1
**(Talc)**	Pouring			245.1	1.2	179.7	1.1	720.7	2.5
**Exp10**	BG	3 (3 × 1 kg)	50	N/A	N/A	N/A	N/A	153.0	1.4
**(Talc)**	Pouring			N/A	N/A	N/A	N/A	651.8	8.8
**Exp11**	BG	0.75 (3 × 0.25 kg)	100	N/A	N/A	N/A	N/A	410.8	1.2
**(Talc)**	Pouring			N/A	N/A	N/A	N/A	1105.5	4.1
**Exp12**	BG	3 (3 × 1 kg)	100	N/A	N/A	N/A	N/A	386.9	1.1
**(Talc)**	Pouring			N/A	N/A	N/A	N/A	7970.2	1.3
**Exp13**	BG	0.75 (3 × 0.25 kg)	25	64.2	1.1	63.7	1.1	78.1	1.5
**(clay OpTiMat)**	Pouring			67.1	1.2	67.7	1.2	435.1	1.1
**Exp14**	BG	3 (3 × 1 kg)	25	35.0	1.1	36.9	1.1	63.9	1.0
**(clay OpTiMat)**	Pouring			38.7	1.4	55.7	1.9	258.3	3.6
**Exp15**	BG	0.75 (3 × 0.25 kg)	50	69.4	1.1	48.0	1.1	99.8	1.1
**(clay OpTiMat)**	Pouring			76.8	1.2	56.4	1.2	409.4	4.5
**Exp16**	BG	3 (3 × 1 kg)	50	61.4	1.1	38.1	1.1	201.9	1.8
**(clay OpTiMat)**	Pouring			54.8	1.1	42.8	1.3	307.6	5.4
**Exp17**	BG	0.75 (3 × 0.25 kg)	100	59.0	1.1	44.1	1.1	97.5	2.1
**(clay OpTiMat)**	Pouring			78.9	1.5	59.8	1.6	1150.6	1.3
**Exp18**	BG	3 (3 × 1 kg)	100	N/A	N/A	54.5	1.1	162.2	1.6
**(clay OpTiMat)**				N/A	N/A	75.6	1.9	868.5	26.1

Table S3. Defined handling energy factors for each experiment by using the data from the SRD and CD dustiness methods. The handling energy factors marked grey with the symbol asterisk (*) resulted from the gravimetrical analysis of the filters collected during the drop tests which might have higher uncertainties (below detection limit or mass that was probably lost during filter handling). N/A: Not available data.

	Amount poured (kg)	Drop height (cm)	$H_{S,\ SRD}$ (Eq.2)		$H_{S,\ CD}$ (Eq.2)		$H_{DIm\ (respirable),\ SRD}$ (Eq.3)		$H_{DIm\ (respirable),\ CD}$ (Eq.3)		$H_{DIm\ (inhalable),\ CD}$ (Eq.3)	
			CPC_{pos1}	CPC_{pos2}	CPC_{pos1}	CPC_{pos2}	Pos1	Pos2	Pos1	Pos2	Pos1	Pos2
**Exp1 (clay Polestar)**	0.75 (3 × 0.25 kg)	25	0	0	0	0	0.03*	0.02*	0.04*	0.03*	0.004	0.01
**Exp2 (clay Polestar)**	3 (3 × 1 kg)	25	0	0.4	0	0.5	0.003*	0.03*	0.004*	0.04*	0.005	0.01
**Exp3 (clay Polestar)**	0.75 (3 × 0.25 kg)	50	0	0	0	0	0.07	0.13	0.09	0.16	0.02	0.03
**Exp4 (clay Polestar)**	3 (3 × 1 kg)	50	2.9	2.0	3.8	2.7	0.10	0.09	0.13	0.11	0.03	0.03
**Exp5 (clay Polestar)**	0.75 (3 × 0.25 kg)	100	0	0	0	0	0.29	0.30	0.37	0.38	0.09	0.09
**Exp6 (clay Polestar)**	3 (3 × 1 kg)	100	0.3	0.6	0.5	0.9	0.31	0.32	0.38	0.40	0.10	0.13
**Exp7 (Talc)**	0.75 (3 × 0.25 kg)	25	0.6	0.0	0.2	0.0	0.07	0.07	0.01	0.01	0.002	0.002
**Exp8 (Talc)**	3 (3 × 1 kg)	25	0.7	0.7	0.2	0.2	0.10	0.10	0.02	0.01	0.003	0.002
**Exp9 (Talc)**	0.75 (3 × 0.25 kg)	50	0.7	0.5	0.2	0.2	0.31	0.28	0.05	0.04	0.01	0.01
**Exp10 (Talc)**	3 (3 × 1 kg)	50	N/A	N/A	N/A	N/A	0.20	0.21	0.03	0.03	0.01	0.01*
**Exp11 (Talc)**	0.75 (3 × 0.25 kg)	100	N/A	N/A	N/A	N/A	0.63	0.66	0.10	0.10	0.02	0.02
**Exp12 (Talc)**	3 (3 × 1 kg)	100	N/A	N/A	N/A	N/A	0.22*	0.39	0.03*	0.06	0.01	0.01
**Exp13 (clay OpTiMat)**	0.75 (3 × 0.25 kg)	25	0.1	0.2	0.2	0.2	0.05	0.04	0.02	0.01	0.02	0.02
**Exp14 (clay OpTiMat)**	3 (3 × 1 kg)	25	0.2	0.9	0.2	1.0	0.06	0.07	0.02	0.03	0.02	0.03
**Exp15 (clay OpTiMat)**	0.75 (3 × 0.25 kg)	50	0.4	0.4	0.4	0.5	0.13*	0.12	0.05*	0.05	0.07	0.07
**Exp16 (clay OpTiMat)**	3 (3 × 1 kg)	50	0	0.2	0	0.3	0.06	0.10	0.02	0.04	0.04	0.05
**Exp17 (clay OpTiMat)**	0.75 (3 × 0.25 kg)	100	1.0	0.8	1.1	0.9	0.26	0.36	0.10	0.14	0.14	0.15
**Exp18 (clay OpTiMat)**	3 (3 × 1 kg)	100	N/A	1.0	N/A	1.2	0.22*	0.15*	0.08*	0.06*	0.09	0.10

Table S4. Outlier test of H_i determined for particle mass and number concentrations for SRD and CD methods considering a normal distribution.

Null hypothesis: All data values come from the same normal population
Alternative hypothesis: Smallest or largest data value is an outlier
Significance level: α = 0.05

Grubbs' Test for SRD (respirable mass):

Variable	Height	N	Mean	StDev	Min	Max	G	P
Hi	25.00	12	0.05331	0.02913	0.00303	0.09615	1.73	0.813
	50.00	12	0.1500	0.0830	0.0617	0.3149	1.99	0.354
	100.00	12	0.3435	0.1563	0.1544	0.6647	2.06	0.275

* NOTE * No outlier at the 5% level of significance

Grubbs' Test for CD (respirable mass):

Variable	Height	N	Mean	StDev	Min	Max	G	P
Hi	25.00	12	0.02020	0.01086	0.00378	0.03985	1.81	0.637
	50.00	12	0.0658	0.0436	0.0235	0.1571	2.09	0.237
	100.00	12	0.1824	0.1484	0.0349	0.3958	1.44	1.000

* NOTE * No outlier at the 5% level of significance

Grubbs' Test for SRD (particle number):

Variable	Height	N	Mean	StDev	Min	Max	G	P
Hi	25.00	12	0.3264	0.3328	0.0000	0.9198	1.78	0.689
	50.00	10	0.720	0.975	0.000	2.915	2.25	0.064
	100.00	7	0.539	0.432	0.000	1.036	1.25	1.000

* NOTE * No outlier at the 5% level of significance

Grubbs' Test for CD (particle number):

Variable	Height	N	Mean	StDev	Min	Max	G	P
Hi	25.00	12	0.2301	0.2897	0.0000	1.0218	2.73	0.004
	50.00	10	0.801	1.331	0.000	3.841	2.29	0.052
	100.00	7	0.628	0.483	0.000	1.151	1.30	1.000

Outlier at 25 cm drop height = 1.0218

Grubbs' Test for CD (Inhalable mass):

Variable	Height	N	Mean	StDev	Min	Max	G	P
Hi	25.00	12	0.00970	0.00868	0.00157	0.02575	1.85	0.564
	50.00	12	0.03115	0.02213	0.00610	0.07208	1.85	0.563
	100.00	12	0.0806	0.0511	0.0114	0.1531	1.42	1.000

* NOTE * No outlier at the 5% level of significance

G: Grubbs' test statistic is the difference between the sample mean and either the smallest or largest data value, divided by the standard deviation.
P: The p-value is a probability that measures the evidence against the null hypothesis. A smaller p-value provides stronger evidence against the null hypothesis.

Table S5. Descriptive Statistics on the calculated H_i for particle mass and number concentrations for both SRD and CD methods. N: number of available data; N*: missing data values.

Statistics for SRD (respirable mass):

Variable	Height (cm)	N	N*	Mean	SE Mean	StDev	Minimum	Q1	Median	Q3
Hi	25.00	12	0	0.05331	0.00841	0.02913	0.00303	0.02976	0.05682	0.07214
	50.00	12	0	0.1500	0.0240	0.0830	0.0617	0.0892	0.1228	0.2096
	100.00	12	0	0.3435	0.0451	0.1563	0.1544	0.2326	0.3036	0.3807

Variable	Height (cm)	Maximum	Skewness
Hi	25.00	0.09615	-0.06
	50.00	0.3149	1.02
	100.00	0.6647	1.32

Statistics for CD (respirable mass):

Variable	Height (cm)	N	N*	Mean	SE Mean	StDev	Minimum	Q1	Median	Q3
Hi	25.00	12	0	0.02020	0.00313	0.01086	0.00378	0.01182	0.01766	0.02797
	50.00	12	0	0.0658	0.0126	0.0436	0.0235	0.0327	0.0471	0.1042
	100.00	12	0	0.1824	0.0429	0.1484	0.0349	0.0646	0.1001	0.3745

Variable	Height_1 (cm)	Maximum	Skewness
Hi	25.00	0.03985	0.50
	50.00	0.1571	1.12
	100.00	0.3958	0.71

Statistics for SRD (particle number):

Variable	Height (cm)	N	N*	Mean	SE Mean	StDev	Minimum	Q1	Median	Q3
Hi	25.00	12	0	0.3264	0.0961	0.3328	0.0000	0.0106	0.1873	0.6604
	50.00	10	2	0.720	0.308	0.975	0.000	0.000	0.387	1.060
	100.00	7	5	0.539	0.163	0.432	0.000	0.000	0.647	0.976

Variable	Height (cm)	Maximum	Skewness
Hi	25.00	0.9198	0.63
	50.00	2.915	1.71
	100.00	1.036	-0.29

Statistics for CD (particle number):

Variable	Height (cm)	N	N*	Mean	SE Mean	StDev	Minimum	Q1	Median	Q3	Maximum
Hi	25.00	12	0	0.2301	0.0836	0.2897	0.0000	0.0033	0.1996	0.2280	1.0218
	50.00	10	2	0.801	0.421	1.331	0.000	0.000	0.243	1.010	3.841
	100.00	7	5	0.628	0.183	0.483	0.000	0.000	0.853	1.084	1.151

Variable	Height (cm)	Skewness
Hi	25.00	2.12
	50.00	1.90
	100.00	-0.50

Statistics for CD (inhalable mass):

Variable	Height (cm)	N	N*	Mean	SE Mean	StDev	Minimum	Q1	Median	Q3
Hi	25.00	12	0	0.00970	0.00251	0.00868	0.00157	0.00249	0.00517	0.01780
	50.00	12	0	0.03115	0.00639	0.02213	0.00610	0.00969	0.03140	0.04587
	100.00	12	0	0.0806	0.0148	0.0511	0.0114	0.0225	0.0902	0.1235

Variable	Height (cm)	Maximum	Skewness
Hi	25.00	0.02575	0.77
	50.00	0.07208	0.66
	100.00	0.1531	-0.20
